# DNA methylation analysis on purified neurons and glia dissects age and Alzheimer’s disease-specific changes in the human cortex

**DOI:** 10.1186/s13072-018-0211-3

**Published:** 2018-07-25

**Authors:** Gilles Gasparoni, Sebastian Bultmann, Pavlo Lutsik, Theo F. J. Kraus, Sabrina Sordon, Julia Vlcek, Vanessa Dietinger, Martina Steinmaurer, Melanie Haider, Christopher B. Mulholland, Thomas Arzberger, Sigrun Roeber, Matthias Riemenschneider, Hans A. Kretzschmar, Armin Giese, Heinrich Leonhardt, Jörn Walter

**Affiliations:** 1 Department of Genetics, University of Saarland (UdS), Campus, 66123 Saarbrücken, Germany; 2 Department of Biology and Center for Integrated Protein Science, Ludwig-Maximilians-University (LMU), 82152 Munich, Germany; 3 Epigenomics and Cancer Risk Factors, German Cancer Research Center (DKFZ), 69120 Heidelberg, Germany; 4 Center for Neuropathology and Prion Research, Ludwig-Maximilians-University (LMU), 82152 Munich, Germany; 5 Department of Psychiatry and Psychotherapy, Saarland University Hospital (UKS), 66424 Homburg, Germany

**Keywords:** DNA methylation, Epigenetics, Alzheimer’s disease, Neurodegeneration, Aging, Cell sorting, Neuron, Glia, Brain, EWAS

## Abstract

**Background:**

Epigenome-wide association studies (EWAS) based on human brain samples allow a deep and direct understanding of epigenetic dysregulation in Alzheimer’s disease (AD). However, strong variation of cell-type proportions across brain tissue samples represents a significant source of data noise. Here, we report the first EWAS based on sorted neuronal and non-neuronal (mostly glia) nuclei from postmortem human brain tissues.

**Results:**

We show that cell sorting strongly enhances the robust detection of disease-related DNA methylation changes even in a relatively small cohort. We identify numerous genes with cell-type-specific methylation signatures and document differential methylation dynamics associated with aging specifically in neurons such as *CLU*, *SYNJ2* and *NCOR2* or in glia *RAI1*,*CXXC5* and *INPP5A*. Further, we found neuron or glia-specific associations with AD Braak stage progression at genes such as *MCF2L*, *ANK1*, *MAP2*, *LRRC8B*, *STK32C* and *S100B*. A comparison of our study with previous tissue-based EWAS validates multiple AD-associated DNA methylation signals and additionally specifies their origin to neuron, e.g., *HOXA3* or glia (*ANK1*). In a meta-analysis, we reveal two novel previously unrecognized methylation changes at the key AD risk genes *APP* and *ADAM17*.

**Conclusions:**

Our data highlight the complex interplay between disease, age and cell-type-specific methylation changes in AD risk genes thus offering new perspectives for the validation and interpretation of large EWAS results.

**Electronic supplementary material:**

The online version of this article (10.1186/s13072-018-0211-3) contains supplementary material, which is available to authorized users.

## Background

Alzheimer’s disease (AD) is a fatal neurodegenerative disorder and the most common form of age-related dementia. The majority of AD cases are diagnosed as late-onset AD but its aetiology is thought to start much earlier in life and to slowly progress until first symptoms emerge [53]. At the cellular level, AD can be characterized by the appearance and extent of (i) extracellular plaques from accumulations of insoluble amyloid beta filaments, (ii) intracellular neurofibrillary tangles of hyperphosphorylated tau [25, 115, 117] and (iii) neuroinflammation. According to the amyloid cascade hypothesis [39], AD results from neurotoxic amyloid beta plaques which form from excess of APP protein filaments derived by sequential proteolytic cleavage by $\beta$- or $\gamma$-secretases [15, 103]. Neuropathologically, AD is classified according to spread of disease marks across the brain regions (Braak stages) [13]. Despite intense research, molecular causes for AD and the relationship to normal aging are not well understood. Only a minority of AD cases can be explained by mutations in the genes *APP*, *PSEN1* and *PSEN2* [16, 22]. Recently, several studies suggest neuroepigenetic mechanisms to be involved in AD etiology suggesting that the sum of acquired epigenetic alterations over lifespan could play a role [68, 88, 89]. In the central nervous system (CNS), these processes are indispensable for cells to execute complex functions such as learning and memory, but are also involved in the context of aging and neuropathologic processes [2, 10, 23, 116]. To date, DNA methylation at cytosines (5-mC) in a CpG sequence context is the best understood and most extensively studied epigenetic mark [75, 76] which can be target for further modifications from 5-mC to 5-hydroxy-methylcytosine, to 5-formylcytosine to 5-carboxymethylcytosine by the family of Tet enzymes [124]. In particular, 5-hmC the most prominent oxidized form of cytosine methylation is identified in cells of the CNS [64] and recent studies in the brain reported altered proportions upon aging and in disease [5, 19, 26, 43, 63, 70, 98, 136].

Two recent large EWAS based on human brain tissue reported Braak stage-associated methylation changes at several genes such as *ANK1*,*RPL13*, *CDH23*, *HOXA3* and *BIN1* [27, 82]. Notably, methylation changes at the *APP* gene locus have so far only been shown for individual AD cases [52, 129, 137] while a larger study could not confirm this observation [6]. One main obstacle is that in the cortex cell-type composition changes upon aging and/or disease progression [122]. This heterogeneity in tissue composition constitutes a major source of noise in epigenetic profiles and compromises a clean distinction of age, cell-type and disease-related changes across samples. Several bioinformatic approaches were developed to address this problem [47, 48, 97, 104]. However, there is an ongoing dispute concerning their proper usability [105, 144]. In our study, we circumvent this problem by profiling cells separated into neuronal- and glia fractions of cortices from 31 healthy donors and AD patients with well-defined Braak stages. A comparison of cell sorting and bulk tissue-based screens reveals that cell-type purification efficiently reduces confounding noise generated by variable cell composition and enhances the detection of disease-related changes. Therefore, we detect a substantial number of Braak stage-associated markers previously only found in much larger datasets of total brain tissue. In addition, cell sorting allows assignment of identified signals to cell-type origin and reveals that next to neurons also glia cells experience strong epigenetic alterations upon AD progression.

## Results

### Cellular heterogeneity in brain tissue undermines the association analysis for Braak stage

The aim of this study is to address DNA methylation changes in the human brain that are associated with aging and/or with increasing Braak stages. Before starting our cell sorting approach, we performed an initial DNA methylation screen on unsorted 128 postmortem bulk brain samples (63 frontal (FC) and 65 temporal cortex (TC) samples) derived from 52 healthy controls (CTRL) and 76 AD donors (Additional file 2: Tables S1–S2) using Illumina’s HumanMethylation 450k bead array platform. Following state of the art data processing  [72] (see "Methods" section), we obtained data for more than 460,000 CpGs per sample. To calculate the association between DNA methylation and Braak stage progression or aging, we applied multiple linear regression models thereby correcting for sex, age and batch effects (see principal component analysis; Additional file 1: Fig. S1k, l). In addition, we calculated for each sample the neuronal content following the method described by Houseman et al. [47]. We separately analyzed FC and TC samples to exclude brain region effects (Additional file 1: Fig. S1a–d) (Additional file 2: Tables S3–S6). With respect to aging, we identified only six differentially methylated CpGs (DMCGs) overlapping between FC and TC when comparing the two top 1000 ranked CpGs (Additional file 1: Fig. S1g) while 171 were common among the top 1000 Braak stage-associated CpG sets (Additional file 1: Fig. S1h). Also, only little overlap was observed for aging and Braak-DMCGs within FC or TC (Additional file 1: Fig. S1e, f). We used the top 1000 top ranking Braak-DMCGs in a cluster analysis, but the samples did not clearly differentiate for diagnosis (Additional file 1: Fig. S1i) and the observed changes were between low and high Braak stages were mostly low (Additional file 1: Fig. S1j) suggesting a limited discriminative power of the analysis. In addition, none of our 200 top ranking CpGs from FC or TC showed overlap with a recently published large AD-EWAS also based on brain tissue material [82]. A possible explanation for this could be that our cohort was smaller compared to the published EWAS. Alternatively, we reasoned that 5-mC profiles might strongly differ between populations (cohorts) or show variable grades of heterogeneity in individual cell-type composition. To overcome this problem, we decided to proceed with a reduced number of samples for which we physically separated neuronal and non-neuronal cell-types.

**Fig. 1:**
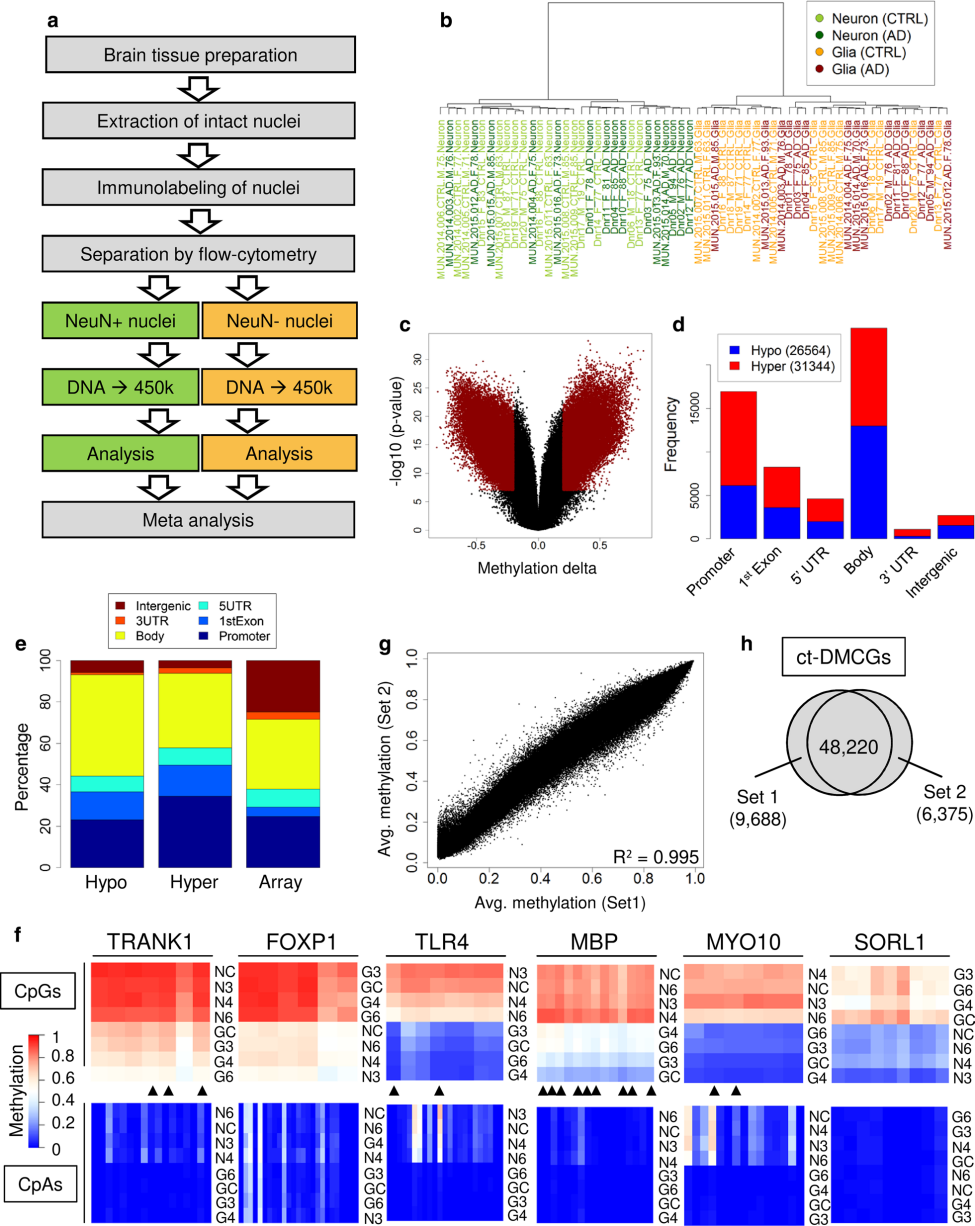
Experimental setup and cell-type-specific DNA methylation signatures. **a** General work flow scheme for neuron and glia cell-type separation and DNA methylation profiling. **b** Cluster analysis on complete data shows full separation of neuron and glia samples. **c** Volcano plot for identification of 57,908 cell-type-specific CpGs (ct-DMCGs, dark red) in sorted CTRLs. **d** Genomic distribution of ct-DMCGs that are hypo- or hypermethylated in neurons. CpGs are classified in respect of hypo-or hypermethylation in neurons compared to glia. **e** Relative distribution of ct-DMCGs in relation to array design. **f** NGS-based validation for exemplary ct-DMCGs in a subset of neuron and glia samples. Columns represent methylation levels of cytosines at CpGs (upper panels) or CpAs (lower panels). Rows are samples ordered by similarity. Black triangles mark CpGs that are present on the 450k array. NC/GC: neuron/glia controls, N3-N6/G3-G6: neuron/glia Braak stages III–VI. **g** Similarity of neuronal methylation profiles (CTRLs only) for our own study (dataset 1) and data from Guintivano et al [35] (dataset 2). **h** Overlap of ct-DMCGs as defined by datasets 1 and 2, respectively

### Cell separation identifies a broad range of cell-type-specific methylation signatures

We performed NeuN immunolabeling followed by FACS separation [3, 51, 55, 80, 93, 100, 128] to obtain neuronal (NeuN-positive, referred to as neurons) and non-neuronal nuclei (NeuN-negative, referred to as glia) from 31 human postmortem occipital cortex samples. From this, we generated 62 epigenome-wide cell-type-specific profiles (Additional file 2: Tables S1–S2) on an Illumina 450k methylation array platform (for general work flow, processing and quality checks see "Methods" section and Fig. 1a). Cluster analysis revealed a clear overall separation of neuronal and glia samples (Fig. 1b). To systematically define cell-type-specific methylation sites (ct-DMCGs), we compared neuron and glia samples (controls only) by a two-sided *t* test and filtered for autosomal CpGs with a significant *p* value (Bonferroni corrected) and methylation difference of at least 20% (Fig. 1c, Additional file 2: Table S7). In total, we identified 57,908 ct-DMCGs (approx. 12% of all sites) distributed across 11,279 genes, many of which are known to be differentially expressed in neurons and glia cells such as *SYNPO* [96] (cg06732545, $p = 7.09 * 10^{-29}$, rank 82), *FOXP1* [125] (cg21836903, $p = 4.48 * 10^{-27}$, rank 329) and *MBP*  [38] (cg24446429, $p = 3.28 * 10^{-22}$). A bit more than half (54%) of all ct-DMCGs were hypermethylated in neurons (Fig. 1d). Most of the ct-DMCGs were located at gene body and promoter regions and only very few in intergenic regions (Fig. 1d). Compared to the array design, we observed more hypomethylated ct-DMCGs at the gene body while hypermethylated ct-DMCGs were enriched at gene promoters and exon 1 regions (Fig. 1e). A GO enrichment analysis revealed mostly neurological process such as ‘neuron projection morphogenesis’, ‘modulation of synaptic transmission’ and ‘regulation of neurotransmitter levels’ (Additional file 2: Table S8). We also validated a larger set of differential loci using deep bisulfite amplicon sequencing (Fig. 1f). We found a high concordance between 450k array data and NGS data ($R^2$ = 0.912; Additional file 1: Fig. S2b, Additional file 2: Table S9). In line with previous findings [75], we detected pronounced non-CpG methylation levels mostly at CpA dyads for neurons and to much a lesser extent for glia (Fig. 1f). In addition, we validated cell-type-specific expression at the protein level using immunohistochemical staining with specific antibodies against *SORL1* and *FOXP1*, respectively (Additional file 1: Fig. S2c, Additional file 2: Table S10). Taken together we confirm a successful and clear separation of neuronal and glia cell fractions from fresh frozen brain tissue.

We next compared our results to a recent study that also applied NeuN-based separation of human neuron and glia cells  [35]. From this dataset, we selected all 23 healthy Caucasian samples (Additional file 2: Table S2). The correlation between our and the Guintivano et al. study was very good ($R^2$ > 0.99) both for the neuronal and glia methylation profiles (Fig. 1g, Additional file 1: Fig. S2d). Moreover, the majority of neuron to glia differences were highly similar (Additional file 1: Fig. S2e) as clearly demonstrated by the concordant placement in a principal component analysis (Additional file 1: Fig. S2a). About 48,220 (88.3%) ct-DMCGs overlapped with those identified in our own data (Fig. 1h). We would like so stress that the Guintivano cohort was derived from frontal cortex while our samples were from occipital cortex. So we wondered if NeuN-sorted samples from different brain regions might exhibit distinct methylation signatures. To follow this question we performed a correlation analysis on a subset of healthy control samples from our cohort (occipital cortex) and from the Guintivano study that are matched for sex, age and cell-type (n = 5). Notably, we observe the same very high degree of correlation within and across cohorts (Additional file 2: Table S11), indicating that NeuN-sorted methylation profiles from both regions are comparable. Further, in a PCA analysis, they clustered together independent of cohort (data not shown). Given this high agreement, we conclude that NeuN-based cell separation from human cortical postmortem samples is a robust and reproducible approach, even across brain regions and cohorts. We therefore felt confident to include the 23 samples from the Guintivano study as additional controls in our subsequent analyses. Other samples from this study including non-Caucasian controls were not used.

**Table 1:** Top 25 aging-DMCGs identified in the meta-analysis

Rank	*P* value (meta-analysis)	*P* value (neuron)	*P* value (glia)	TargetID	Chr	Position	Gene	Region	FDR
1	4.34E−27	6.58E−09	1.00E−20	cg13327545	10	22623548			2.0763E−21
2	9.38E−26	5.55E−10	2.69E−18	cg16867657	6	11044877	ELOVL2	TSS1500	2.2438E−20
3	2.53E−25	8.99E−09	4.56E−19	cg20224218	9	129261375	FAM125B	Body	4.0346E−20
4	4.38E−25	5.03E−06	1.42E−21	cg10804656	10	22623460			5.2387E−20
5	6.46E−25	9.09E−14	1.17E−13	cg14919554	5	43018629			6.1811E−20
6	8.12E−25	2.95E−17	4.54E−10	cg02426178	19	10747142	SLC44A2	Body	6.4746E−20
7	1.65E−24	5.49E−16	5.01E−11	cg03984866	4	8161776	ABLIM2	TSS1500	1.0465E−19
8	1.75E−24	1.52E−09	1.92E−17	cg10906284	12	63544430	AVPR1A	1stExon	1.0465E−19
9	2.38E−24	2.13E−06	1.88E−20	cg17117277	19	3822126	ZFR2	Body	1.2651E−19
10	5.41E−24	2.75E−08	3.36E−18	cg06022942	10	8095484	FLJ45983	TSS200	2.5882E−19
11	7.95E−24	3.53E−14	3.86E−12	ch.6.1693624F	6	83767401	UBE2CBP	Body	3.4576E−19
12	1.32E−23	5.62E−09	4.06E−17	cg24954207	3	128217091			5.2626E−19
13	1.77E−23	4.00E−09	7.68E−17	cg08234504	5	139013317			6.5138E−19
14	1.92E−23	8.17E−11	4.10E−15	cg02492920	7	150659969	KCNH2	Body	6.5384E−19
15	2.05E−23	1.01E−11	3.53E−14	cg14299508	8	105907690			6.5384E−19
16	3.75E−23	2.50E−12	2.64E−13	cg08594681	8	27468684	CLU	1stExon	1.1213E−18
17	4.63E−23	6.45E−14	1.27E−11	cg08342886	6	33240066	VPS52	TSS1500	1.303E−18
18	1.20E−22	9.22E−10	2.34E−15	cg00303378	1	159825552	VSIG8	Body	3.0971E−18
19	1.23E−22	1.21E−10	1.83E−14	cg23813012	1	14026482	PRDM2	TSS1500	3.0971E−18
20	1.38E−22	5.01E−10	4.98E−15	ch.13.22912778R	13	24014778			3.2806E−18
21	1.44E−22	1.20E−08	2.17E−16	cg06639320	2	106015739	FHL2	TSS200	3.2806E−18
22	1.77E−22	1.31E−09	2.45E−15	cg15393490	1	207996459			3.8491E−18
23	1.90E−22	6.33E−08	5.46E−17	cg26880525	1	209877941	HSD11B1	5UTR	3.8672E−18
24	1.94E−22	6.83E−13	5.17E−12	cg04211309	16	58056296			3.8672E−18
25	2.33E−22	5.12E−15	8.29E−10	cg09131339	1	109914235	SORT1	Body	4.4588E−18

**Table 2:** Top 10 Braak stage DMCGs plus 25 selected CpGs from 200 top entries

Rank	*P* value (meta-analysis)	*P* value (neuron)	*P* value (glia)	TargetID	Chr	Position	Gene	Region	Probe SNPs	FDR
1	1.50E−13	1.15E−06	3.83E−09	cg06549928	1	89990868	LRRC8B	5UTR		7.17624E−08
2	6.43E−11	4.64E−06	4.98E−07	cg21913630	7	128828599	SMO	TSS200		1.53811E−05
3	2.05E−10	3.04E−05	2.54E−07	cg16562251	1	166845621	TADA1	1stExon		3.26918E−05
4	2.90E−10	1.15E−05	9.63E−07	cg08738571	12	32655192	FGD4	1stExon	rs56168193	3.45416E−05
5	3.61E−10	9.00E−06	1.54E−06	cg22140756	2	177895827				3.45416E−05
6	4.81E−10	7.42E−06	2.52E−06	cg04913265	11	133939627	JAM3	Body		3.8353E−05
7	8.78E−10	1.69E−05	2.07E−06	cg20693608	4	113152836	AP1AR	TSS200		6.0007E−05
8	1.57E−09	7.82E−06	8.20E−06	cg24449302	15	66679100	MAP2K1	TSS200	rs77540803	7.02674E−05
9	1.60E−09	7.69E−06	8.51E−06	cg13172549	7	27153636	HOXA3	5UTR		7.02674E−05
10	1.61E−09	2.63E−06	2.50E−05	cg04547723	14	75421960	PGF	5UTR		7.02674E−05
11	1.67E−09	1.49E−05	4.58E−06	cg07835289	1	236030215	LYST	5UTR		7.02674E−05
15	2.27E−09	0.00021772	4.33E−07	cg11817993	14	92572978	ATXN3	TSS200		7.02674E−05
21	5.19E−09	3.14E−05	7.12E−06	cg02037503	14	23540729	ACIN1	1stExon		0.000118237
27	1.04E−08	7.43E−05	6.24E−06	cg03040740	13	99084682	FARP1	Body	rs6491426	0.000184279
32	1.44E−08	0.00087784	7.40E−07	cg13859639	11	2846716	KCNQ1	Body		0.000215287
38	2.41E−08	0.00041341	2.70E−06	cg03907612	6	41703314	TFEB	5UTR		0.000303416
39	2.60E−08	7.11E−05	1.70E−05	cg16568373	17	4853368	ENO3	TSS1500		0.000318944
48	3.48E−08	2.84E−05	5.76E−05	cg05884192	14	52515736	NID2	Body	rs2516585	0.000334891
53	3.75E−08	0.00012506	1.42E−05	cg08883485	1	201619787	NAV1	Body		0.000334891
58	4.55E−08	4.14E−05	5.24E−05	cg23712970	14	23540735	ACIN1	1stExon		0.000374624
74	6.71E−08	1.05E−05	0.0003106	cg08843538	2	210288784	MAP2	1stExon		0.000429299
76	6.94E−08	4.79E−05	7.07E−05	cg04542489	14	23775746	BCL2L2	TSS1500		0.00043306
102	1.24E−07	4.82E−05	0.00012891	cg06355720	1	153333350	S100A9	3UTR	rs743566	0.000581604
103	1.27E−07	5.37E−05	0.00011905	cg26327118	6	39693366	KIF6	TSS200		0.000589892
104	1.31E−07	1.08E−05	0.00061189	cg16258854	2	20648194	RHOB	1stExon	rs1062292	0.000595763
111	1.41E−07	0.00052315	1.36E−05	cg08866780	21	27543523	APP	TSS1500		0.000604315
112	1.42E−07	0.01466411	4.90E−07	cg03613822	17	7115140	DLG4	Body		0.000604315
126	1.71E−07	5.41E−06	0.00161641	cg06291595	14	74960292	NPC2	TSS1500		0.000646609
131	1.86E−07	0.01071281	8.90E−07	cg04153489	8	41655983	ANK1	TSS1500		0.000679278
133	1.99E−07	2.07E−05	0.00049571	cg19447671	2	176032513	ATF2	5UTR		0.000715825
134	2.04E−07	0.00058734	1.79E−05	cg10313337	16	68823690	CDH1	Body		0.000728335
141	2.18E−07	0.00020684	5.47E−05	cg01015899	12	120663812	PXN	5UTR		0.000737839
152	2.41E−07	2.45E−05	0.00051347	cg27212541	3	49507385	DAG1	TSS200		0.00075801
161	2.68E−07	0.00027187	5.17E−05	cg07745886	8	42150794	IKBKB	Body		0.000796369
199	4.19E−07	0.00095868	2.35E−05	cg01231165	2	9695142	ADAM17	Body		0.001001966

### Neuron and glia methylomes change distinctly upon aging

**Fig. 2:**
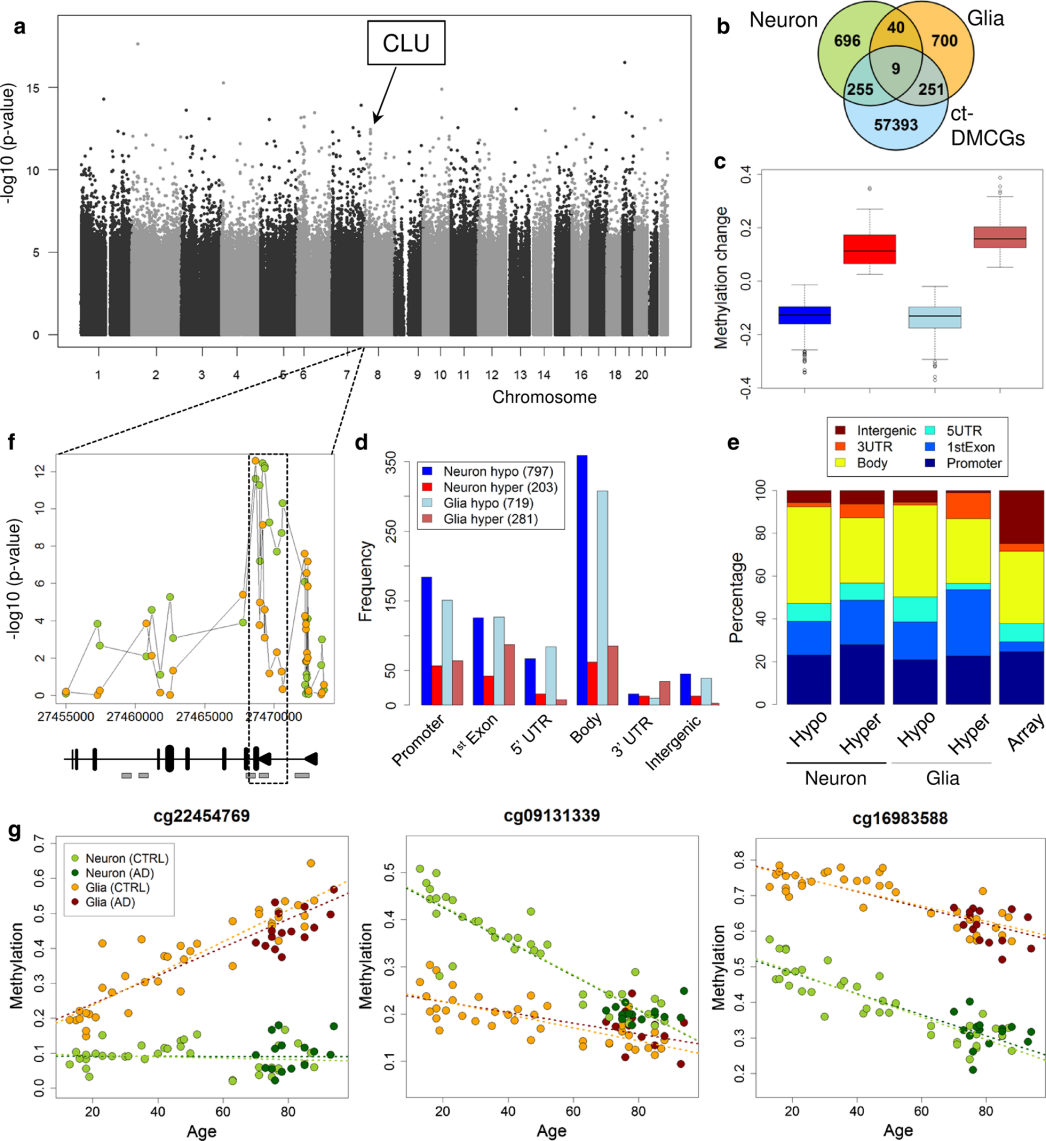
Aging analysis in neurons and glia. **a** Genomic *p* value distribution for the aging analysis in neurons. The arrow marks a region on chromosome 8 with a concentrated set of top-ranking CpGs (see **f**). **b** Overlap of top 1000 aging-DMCGs between neuron and glia, but approx. 25% of each set are ct-DMCGs. **c** Methylation change from 8 youngest to 8 oldest samples for top 1000 aging CpGs in neurons and glia, respectively. Boxes colored as in **d**. **d** Genomic region classification of top 1000 aging-DMCGs from neuron and glia, respectively. CpGs are classified in respect of hypo- or hypermethylation in 8 oldest versus 8 youngest CTRL samples. **e** Relative distribution of top 1000 aging-DMCGs in relation to array design. **f** *p*-value distribution at the clusterin gene locus (*CLU*) in the age analysis in neuron (green) and glia (red), respectively. A set of 10 adjacent CpGs (dashed square) shows strong age-association in healthy neurons (but only partially in glia). Numbers on x-axis give coordinates on chromosome 8 (hg19). Cartoon below illustrates the relative location of exons (black boxes), promoters (black triangles) and annotated sites of high DNaseI hypersensitivity (gray boxes) according to UCSC genome browser. **g** Age-dependent methylation changes that occur exclusively in one cell-type can lead to either emergence (left panel) or disappearance (mid) of significant differences between cell-types. Aging effects that are synchronized between cell-types are stable ct-DMCGs over lifetime (right). Adding AD samples leads to similar results (bright dashed lines: regression lines based on CTRLs, dark dashed lines: CTRLs + AD cases) (color figure online)

To analyze age-associated DNA methylation changes in both cell fractions (Table 1) we applied multiple linear regression models separately for the neuron and glia samples correcting for the confounding variables sex and technical batch effects (see "Methods" section). While we detected many aging-DMCGs with large methylation differences between young and old individuals (Fig. 2a, c; Additional file 1: Fig. S2a) there was only a minor overlap ($n = 49$) of the top 1000 aging-DMCGs between neurons and glia (Fig. 2b), indicating distinct methylation dynamics for both cell-types upon aging (Additional file 2: Tables S12, S13). Most aging-DMCGs were found in gene body, promoter and first exon regions while intergenic regions were underrepresented (Fig. 2d, e). For both cell-types, we predominantly observe a hypomethylation with increasing age (Fig. 2d, Additional file 1: Fig. S3). For several genes in the top 1000 lists, we observed prominent age-associated changes in neurons such as *FAM53B* (10 hits), *CLU* (8) and *NCOR2* (6) (Additional file 2: Table S14). All the three genes encode proteins important for neuronal processes  [36, 54, 60]. Interestingly, the aging-DMCGs at the *CLU* gene are specifically concentrated in the first intron (Fig. 2f) at a previously described alternative promoter where a specific CLU isoform is initiated from [11, 74]. These CpGs show a strong loss of DNA methylation with increasing age in neurons (Additional file 1: Fig. S4). The effect is independent of AD as we observe this in healthy controls and AD samples to the same extent. In nuclei from glia, we confirmed aging-DMCGs at loci which were previously described as aging markers in peripheral blood such as *ELOVL2* and *FHL2* [59]. In addition, we identified a set of new genes with clustered and prominent aging-DMCGs glia such as *RAI1* (6 hits), *CUX1*, *DIP2C*, *FLJ45983* and *ITPK1* (all 5) (Additional file 2: Table S14). For both neurons and glia, the top ten aging-associated KEGG pathways featured ErbB signaling pathway, Neuroactive ligand-receptor interaction and MAPK signaling pathway (Additional file 2: Table S19). Notably, we found numerous aging-DMCGs with asymmetric or even opposing methylation dynamics in neurons and glia, respectively (Fig. 2g, Additional file 1: Fig. S5). Such cases nicely illustrate the complexity of aging across cell-types and the advantage of cell sorting over bulk tissue.

Among genes with multiple age-dynamic ct-DMCGs (Additional file 2: Table S15) we identify prominent candidates with negative cell-type-specific aging dynamics (i.e., neuron-glia differences are decreased in old individuals compared to young ones) such as *C21orf34* (6 hits), *ANK1* (5), *PTPRN2* (5), *HDAC4* (5), but also *APP* (2).

*ANK1* was recently reported as a top differentially methylated marker associated with Braak stage progression in two large EWAS [27, 82]. Indeed we find that the two top *ANK1* CpGs and several others reported in these studies exhibit strong and complex cell-type dynamics overage (Additional file 1: Fig. S6). Increased cell-type-specific aging dynamics were also seen in *DIP2C5* (5) and *PAX6* (5). The latter encodes an transcription factor that is important in the development processes of neural tissues and has been implicated in healthy aging and AD [81].

Next, we independently validated our top aging-DMCGs using another public cohort of NeuN-sorted prefrontal cortex brain samples [61]. From this dataset (GSE98203), we only used healthy, NeuN-positive controls as NeuN-negative data was not available. For our top 50 neuronal aging-DMCGs we found highly similar methylation dynamics overage in these samples (examples see Additional file 1: Fig. S7) illustrating that our approach is valid. These findings may have profound implications for the interpretation of epigenetic effects in age-related diseases. In many studies, bioinformatic methods are used to adjust tissue samples for cell composition effects [46–48, 104] which is inferred from reference datasets. In the light of our observations, this procedure could be problematic if the used references and tissue samples have a different age distribution because the tool potentially selects discriminative markers that have diverse aging dynamics. Indeed, our *in silico* estimations of neuronal content in brain tissue show systematic differences depending on the usage of a reference set of young age or of old age (Additional file 1: Fig. S8a, b). In addition to that we find high variation of neuronal content across all studies and tissues analyzed (Additional file 1: Fig. S8c). Our analysis emphasizes that cell-type-specific methylation dynamics upon aging are prominent and have to be considered as complex confounding factors in epigenetic studies of brain tissues.

### Both neurons and glia show Braak stage-associated methylation changes

**Fig. 3:**
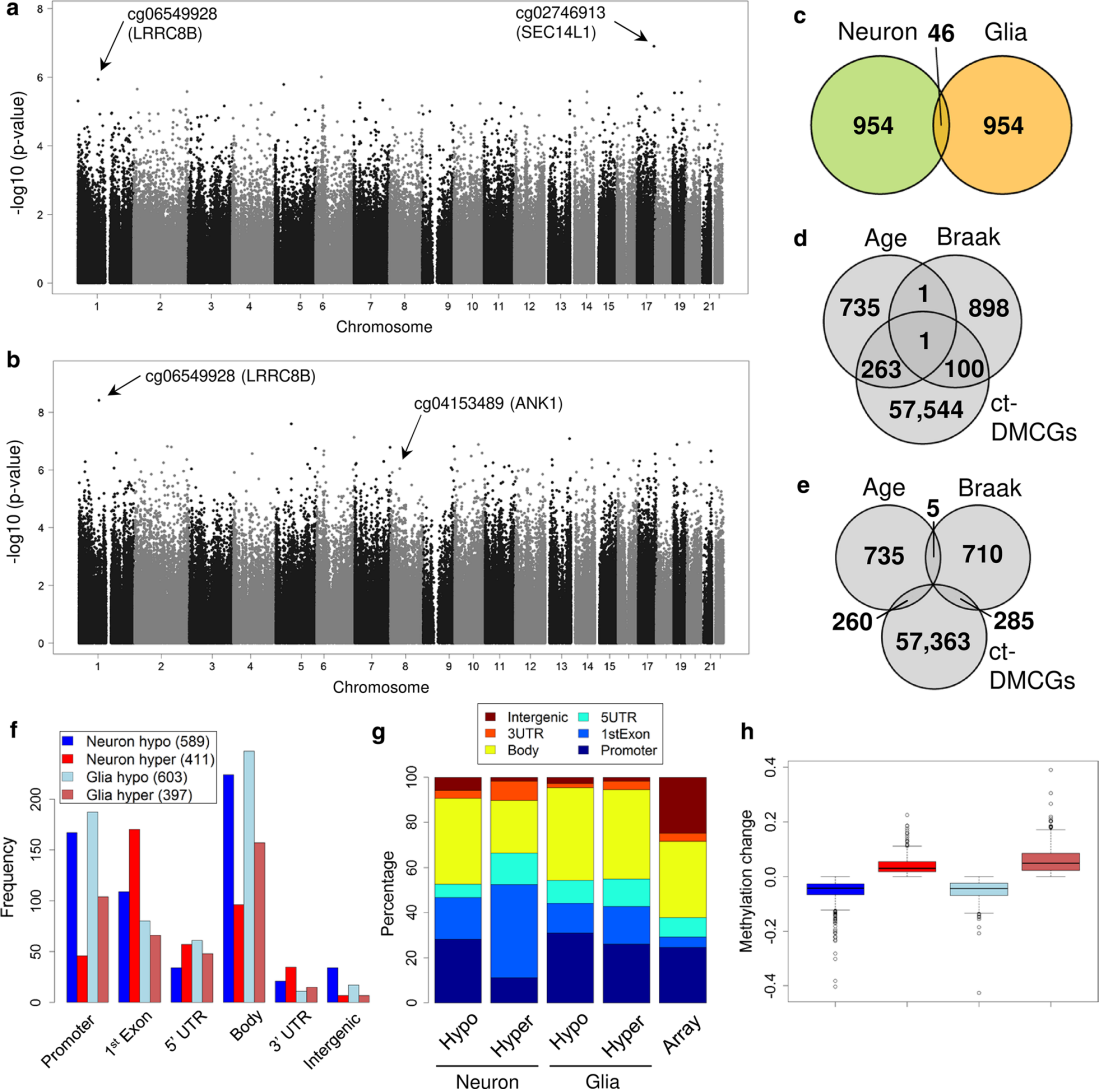
Braak stage analysis in neuron and glia. **a** Genomic *p* value distribution for the Braak stage progression analysis in neurons. **b** Genomic *p* value distribution for the Braak stage progression analysis in glia. **c** Overlap for the top 1000 ranking Braak-DMCGs from neuron and glia. **d** Overlap of top 1000 Braak-DMCGs and top 1000 aging-DMCGs from neuron, 10% of Braak-DMCGs are ct-DMCGs. **e** Same as **d** but for glia top DMCGs. Compared to neuron there are more ct-DMCGs among top Braak-DMCGs in glia. **f** Distribution across genomic regions of top 1000 Braak-DMCGs from neuron and glia. CpGs were classified as hypo- or hypermethylated in respect to methylation change from 8 youngest CTRLs to 8 oldest Braak stage VI samples. Most methylation changes are at gene body, promoters and 1st exon regions. **g** Similar to **f** but in relation to array design. **h** Methylation changes from 8 youngest CTRLs to 8 oldest Braak stage VI samples for top 1000 Braak-DMCGs in neurons and glia, respectively. Boxes colored as in **f** (color figure online)

**Fig. 4:**
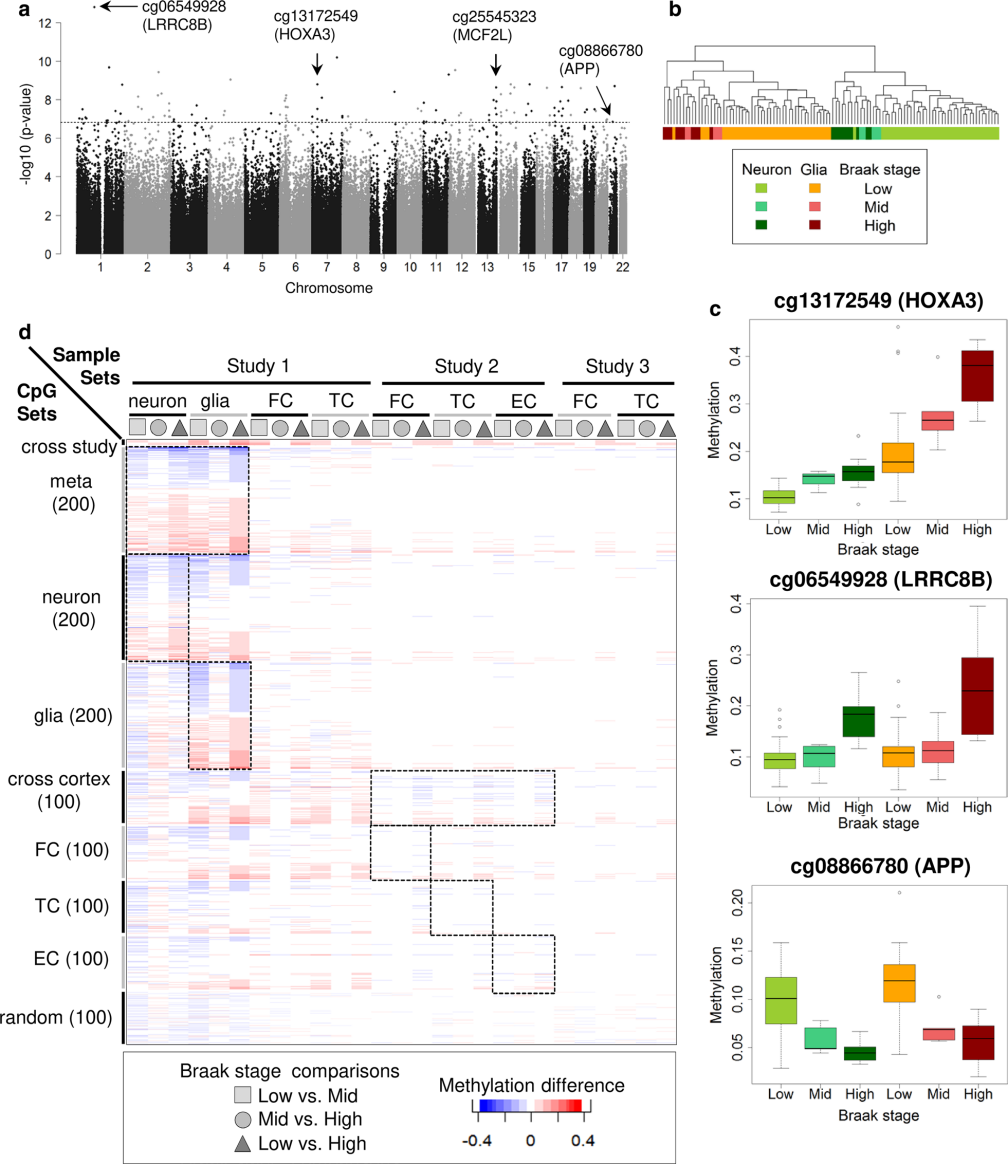
Meta-analysis and integration of external brain tissue data. **a** Manhattan plot for the Braak stage meta-analysis. Selected CpGs located at the genes *LRRC8B*, *HOXA3* and *APP* are marked by arrows. **b** Cluster analysis for the 200 top-ranking Braak-DMCGs. **c** Methylation change over Braak stages for exemplary top CpGs at the genes *HOX3A* (top), *LRRC8B* (middle) and *APP* (bottom). Box colored as in **b**. **d** Methylation change between Braak stages for various CpG sets based on our own data (Study 1: our sorted and tissue samples) or on external tissue data (Study 2: GSE59685 [82]; Study 3: GSE80970 [121]). Rows are organized as sets of CpGs derived from our own 200 top-ranking results (neuron, glia, meta-analysis), the top 100’s from Lunnon and partners (FC, TC, EC, CER) and 100 randomly selected sites. Dashed squares indicate the dataset initially used to identify the corresponding CpG set. ‘Cross study’ depicts two CpGs at the *HOXA3* locus co-identified in our screen and in Study 2. Note that for most CpGs the methylation difference across Braak stages are strongest in sorted samples even when the CpG set was originally identified in tissues. (FC: frontal cortex, TC: temporal cortex, EC: entorhinal cortex) (color figure online)

We then focused on the association of DNA methylation with Braak stage progression in neurons and glia cells, respectively (Fig. 3a, b, Table 2). Similar to the previous analysis we analyzed neuron and glia separately and applied regression models that used the samples’ individual Braak score as a factor. In addition, we corrected for potential confounding variables (aging, sex and batch effects). Again, we observe reduced overlap between the top 1000 Braak-DMCGs of both cell-types (Fig. 3c) indicating that neuronal and glia cells respond differentially during AD progression. For numerous Braak-DMCGs we could observe unbalanced or even reciprocal methylation dynamics in neurons and glia upon Braak stage progression (Additional file 1: Fig. S9). These diametric methylation changes in the two major cell-types of the brain could not be detected in bulk brain tissue screens but rather be interpret as changes in cell proportions. Similarly, the overlap between top Braak-DMCGs and aging-DMCGs is small (Fig. 3d, e), suggesting differential methylation dynamics for aging and Braak stages. Approx. 60% of DMCGs become hypomethylated with increasing Braak stages (Additional file 1: Fig. S10). They are mostly located in gene body, promoters and first exon regions (Fig. 3f) while intergenic regions were largely underrepresented (Fig. 3g). Absolute methylation differences for the top 1000 Braak-DMCGs remained small but clearly higher than the overall changes seen in tissue-based analyses (Fig. 3h, Additional file 1: Fig. S1j). The top ranked neuronal Braak-DMCGS are located at genes involved in cortical neurotransmitter transport. For example, the top-ranking CpG, “cg02746913” ($p \hbox {value}: 1.23 * 10^{-07}$; Additional file 2: Table S16) is located proximal to the gene *SEC14L1* [108]. The top 3 ranking site, “cg06549928” ($p \hbox {value}: 1.15 * 10^{-06}$, Fig. 4c, Additional file 2: Table S16) is located at the *LRRC8B* locus [87]. In addition, we identify clusters of DMCGs in genes such as *MCF2L* (7 hits), *FAM83H* (6), *ARSG* (3) and *HOXA3* (3) (Additional file 2: Table S14).

The glial top marker “cg06549928” ($p \hbox {value}: 3.83 * 10^{-9}$; Additional file 2: Table S17) in the gene *LRRC8B* was also found to be prominent in our neuron analysis. Clustered glia DMCGs were associated with *DIP2C* (6 hits), *HLA-DPB1* (6), *GNG7* (5) and *HOPX* (5) (Additional file 2: Table S14). *GNG7* encodes for a calcium-dependent G coupled receptor that is coupled to dopamine receptors and its expression level in astrocytes becomes upregulated upon inflammatory stimuli [24, 37, 112, 114]. In our list of top glial DMCGs we further identify two hits in the *ANK1* gene (Additional file 2: Tables S14, S17) recently reported as the top marker in two large AD screens on bulk tissue [27, 82]. Our data suggest that the reported epigenetic changes in *ANK1* are restricted to glia.

### Meta-analysis for Braak stage progression detects robust methylation changes in *HOXA3*, *APP* and *ANK1*

Next, we reasoned that a combination of the cell-type-specific results from the neuron and glia analyses should reflect the combined epigenetic burden in the CNS upon Braak stage progression. The combined meta-analysis was performed using Fisher’s Method and results were ranked according to *p* values (Fig. 4a, Table 2). As a plausibility check for our approach, we performed a cluster analysis of the 200 top-ranking combined Braak-DMCGs using all samples. The clustering shows an almost perfect cell-type-specific split on the first level but on the second or third split disease-related sub-clusters (Fig. 4b). In our top 200 list (Additional file 2: Tables S14, S18) the most covered KEGG pathways were ‘MAPK signaling’, ‘neurotrophin signaling’ and ‘Alzheimer’s disease’ (Additional file 2: Table S19). We identified multiple hits for the *HOXA3* gene with a high significance for four CpGs (‘cg13172549’, ‘cg07061298’, ‘cg00921266’, ‘cg22962123’; ranks: 10, 44, 91 and 158). All four CpGs show a solid hypermethylation upon Braak stage progression with more pronounced changes in glia (Fig. 4c, Additional file 1: Fig. S5). Two of these four DMCGs *HOXA3* were recently reported in a large EWAS screen by Lunnon et al [27, 82] analyzing several hundred samples. To systematically examine the overlap between our top 200 markers with all published large tissue-based EWAS studies we retrieved the methylation data from publicly available tissue-based AD datasets (GSE59685, GSE80970). Together with our own dataset this meta-analysis comprised about 700 samples from three brain regions (frontal, temporal, occipital and entorhinal cortex). Following a uniform data processing, we calculated absolute methylation changes between low, middle and high Braak stages for the individual top CpGs (Fig. 4d). We compared the changes associated with Braak progression for our top Braak-DMCGs (top 200 neuron, top 200 glia, top 200 combined analysis) and the top 100 lists from tissue-based EWAS (Fig. 4d). This confirmed two of the four *HOXA3* gene markers across all studies and a remarkable overlap for many more previously reported DMCGs (Fig. 4d, Additional file 1: Fig. S8). For the majority of these DMCGs methylation changes were more pronounced in sorted cells than in bulk samples (Additional file 1: Fig. S12) and interestingly often found in glial cells (e.g., *ANK1*). Notably, among the novel Braak-DMCGs in our top 200 DMCGs we found a marker near the *APP* gene (cg08866780, $p = 1.41 * 10^{-7}$, rank 111, Fig. 4c, Additional file 2: Table S18) one of the most relevant AD genes  [39, 115]. The DMCG is located in the *APP* promoter and becomes hypomethylated during Braak stage progression in neurons and in glia but starting from different basal levels (Fig. 3c). This CpG did not show significant changes upon aging in controls only (neuron: $P = 0.4$, glia: $p = 0.46$) and the effect remained when only individuals older than 64 years were considered (Additional file 1: Fig. S11a) suggesting an age-independent methylation change at the *APP* promoter linked to Braak stage. Methylation changes for *APP* were not detected in our bulk brain tissue samples (Additional file 1: Fig. S11b) nor reported in the top 100 lists from tissue screens [27, 82] indicating a gain of sensitivity for pre-sorted samples.

## Discussion

Our first systematic cell-type-specific DNA methylation screen on a cohort of postmortem cortex samples shows that cell sorting strongly enhances the detection of DNA methylation markers and allows a deep insight into cell-type-specific dynamics in the context of aging and AD Braak stage progression, respectively. In general, we observe a deep interplay between DNA methylation changes associated with aging, with cell-type and with disease progression. Many CpGs reveal distinct methylation dynamics during aging in neurons and glia, respectively. This suggests that the biological “clock” of neuronal and glial cells show characteristic epigenetic signatures in the brain. Nevertheless, we also found CpGs with ‘synchronized’ dynamics across the two cell-types (such as in the pan-aging marker *ELOVL2*). In the context of a highly age-related and complex disease such as AD, it is of great value to better understand epigenetic effects linked to aging as it supports the interpretation of molecular changes. Starting off with a conventional tissue-based screen of unsorted cortical tissues from a representative cohort of 128 well diagnosed healthy donors and AD patients we realized that this approach comes with a low detection power and consequently a minimal number of epigenetic markers which moreover mostly do not overlap with other large EWAS studies. To overcome this obstacle we developed a sorting protocol for neuronal and glial nuclei fractions and performed two simultaneous 450K analyses per donor. In line with recently published data [35], we find tens of thousands robust methylation changes between the neuronal and glial fractions. This strong overlap and the high similarity of samples (age- and sex-matched) between the two studies illustrate the high reproducibility of the cell sorting approach. By using linear regression models we then identified robust cell-type-specific DNA methylation differences associated with either aging or Braak stage progression (or both). While age-associated changes have been reported previously on mixed tissues [58, 118] our study highlights the presence of cell-specific age-related methylation dynamics in human primary cells such as neuron and glia. This finding is well compatible with findings reported by others [122] who found age-specific transcriptional differences between neuron and glial cells in the human brain. As one prominent example, we identify a cell-type-specific cluster of age-associated DMCGs located in an alternative promoter of the clusterin *CLU*, a well-known AD risk gene  [8, 30, 40, 67, 69, 94, 94, 110, 138, 141, 143, 145]. At this promoter, neurons become increasingly hypomethylated over lifetime—while changes in glia are only moderate or absent. This effect is independent of AD diagnosis indicating that the epigenetic changes in CLU are a sign of “natural aging” contributing to disease aetiology. In the human two distinct CLU isoforms have recently been described with opposing functions in cell survival and apoptosis [21, 28, 73, 130, 131]. Our data indicate that an alternative promoter becomes epigenetically “activated” in aged neurons and the expression of a pro-apoptotic CLU isoform may contribute to degeneration predominantly in the CNS. Our observation that over lifetime cell-type-specific DMCGs can emerge or vanish indicates that besides cell composition age represents a complex confounding parameter in tissues based EWAS. Our studies show that such phenomena require strong attention particularly when using cell-type-specific data as references for calculation of cell-type proportions and adjustments. We believe that our analysis combined with data from Guintivano et al. represents a valid resource to appropriately identify and interpret age-related effects for data deconvolution. Moreover, we were able to replicate our top neuronal aging-DMCGs in an independent dataset which nicely illustrates that our findings are reproducible. The main goal of our study was to identify DMCGs associated with Braak stage progression on a cell-type-specific basis. In line with earlier studies, we detected DMCGs in several genes involved in neurotransmitter homeostasis and transport such as *SEC14L1*, *MCF2L* and *LRRC8B* [41, 87, 108]. In AD, neurotransmitter systems are largely disturbed [32, 90] and it is known that ACh-releasing neurons are explicitly vulnerable to amyloid beta 1–42 exposition and predominantly lost upon Braak stage progression [4, 99, 113]. In line with this interpretation, we find epigenetic changes in three genes. *MCF2L* encodes a protein involved in the Mcf2l-RhoA-ROCK signaling pathway that mediates Il1rapl1-dependent formation and stabilization of glutamatergic synapses of cortical neurons [41]. *SEC14L1* encodes a protein involved in vesicular trafficking of acetylcholine (ACh) to synaptic vesicles [108]. The LRRC8 genes encode for anion channels that transport the neurotransmitters glutamate, aspartate and gamma-aminobutyric acid (GABA) and the LRRC8B protein has been reported to modulate the substrate specificity of the channels  [87]. Most intriguingly the very prominent changes for *LRRC8B* in both neuronal and glial cells indicate that this anion channel gene plays a multiple role in the AD pathology. Among the many DMCGs changing in glial cells upon Braak stage progression, we found two prominent CpGs in the *ANK1* gene, a gene recently reported as a top candidate in two large EWAS based on hundreds of bulk tissue samples [27, 82]. Our finding underlines that these top markers predominantly change in glia cells. Indeed, a recent study using laser microdissection reported increased *ANK1* expression in AD microglia but not in neurons or astrocytes [92]. To evaluate the neuronal and glial “epigenetic burden” in the AD brain upon Braak stage progression we performed a meta-analysis combining the data of significant “clean”, i.e., cell separated, DMCGs from glia and neuron. This combined analysis validates a series of concurrent changes in known AD genes such as *APP*, *HOXA3* and *ADAM17* and confirms potentially new AD loci such as *LRRC8B* and *MCF2L*. The detection of a Braak stage-associated epigenetic change in the *APP* gene is of particular importance. To our knowledge, we report the first systematic analysis confirming a few unlinked single case reports [52, 129, 137] for *APP*. This DMCG is located at the promoter overlapping with a confirmed CTCF binding region (Additional file 1: Fig. S11c). This region has been shown to regulate APP transcription [133, 134, 142]. Our data suggest that during AD progressive loss of DNA methylation at this region results in enhanced binding of CTCF thus increasing APP transcription. Our analysis further identifies six CpGs high ranking Braak-DMCGs at the *HOXA3* gene. Several of them were previously reported by recent large EWAS studies  [27, 82, 121]. With the help of cell sorting, we could detect these signals in a much smaller cohort illustrating the gain of sensitivity after separation of major cell-types. The HoxA gene cluster is known to coordinate neuronal development, organization of neural circuits and regulation of postmitotic neurons  [79, 91, 101, 135] and abnormal expression or epigenetic dysregulation of Hox genes has been reported for several neurological diseases such as Huntington’s disease, Parkinson’s disease, C9ORF72-related dementias and glioblastoma [31, 45, 65, 66, 78, 139].

There are several limitations to our work. We used classic bisulfite conversion chemistry in our study that is known to be masked for 5-mC and 5-hmC. Future work could employ methods, such as oxidative or Tet-assisted bisulfite conversion to elaborate alterations of individual methylation forms. Further, for AD an predominant loss of pyramidal neurons has been reported [12]. Therefore, some of our results might reflect a shift in neuronal subtype proportions. Additional sorting of neuronal and glial fractions into subpopulations such as dopaminergic, glutamatergic and GABAergic neurons or glial subtypes (astrocytes, oligodendrocytes and microglia) could further enhance the sensitivity of such screens like ours. Indeed combined FACS separation with NeuN and SOX6 has been shown to effectively separate glutamatergic and GABAergic neurons in mouse and identified clear additional neuronal epigenetic differences [62]. However, to date, only these two markers are available and have not been successfully used for postmortem human tissues probes. An alternative approach to dissect the nature of heterogeneity by single nuclei analyses. Such promising approaches have recently been applied on single NeuN-positive nuclei in mouse and human [84], but are still not in a robust state for large-scale comparative studies on challenging postmortem material. Moreover, we included combined nuclei sorted from occipital cortex and frontal cortex in our study to increase sample size. While in brain tissue-based screens strong methylation differences across brain regions have been shown [26, 43], this was so far not observed in sorted NeuN-negative (glia) nuclei [109]. For NeuN-positive samples limited methylation changes were found only between certain regions [109]. Further studies are required to systematically dissect neuronal methylation differences across and within specific brain regions, especially in the cortex. However, in our Braak association analysis, we do not see an enrichment of cell-type markers exclusively found in frontal cortex or in occipital cortex (Additional file 2: Table S21).

## Conclusions

We demonstrate the application of our approach requires a relatively small cohort to robustly identify new and assign known disease-associated epigenetic signals to specific cell populations. We conclude that sorting for major cell-types enables systematic EWAS with less samples compared to common tissue-based screens reducing time, costs and labor. Taken together, our study highlights cell-type composition and aging as major confounding factors in complex tissue analyses and strongly recommends to conduct EWAS on pre-sorted cell-types.

## Methods

### Human brain tissue samples and neuropathological classification of AD

For our study, we used snap-frozen human postmortem cortical tissue from 159 individuals that donated their brains for research (Additional file 2: Tables S1, S2). For 128 frontal and temporal cortex samples, we used the bulk tissue while for the 31 occipital cortex samples (Brodmann Area 17–19) we isolated neuronal and glial nuclei populations. Samples were provided by the Neurobiobank Munich (NBM) and are well characterized by age, gender, disease history and neuropathological findings including Braak stage measurements in the analyzed subregions. All cases were collected according to the NBM standard protocols established by the BrainNet Europe and BrainNet Germany. Written informed consent was obtained according to the guidelines of the local ethics committee. Besides samples stored at $-80\,^{\circ }\hbox {C}$ for molecular analysis some parts of the brains were formalin-fixed and paraffin-embedded (FFPE) for detailed anatomical and neuropathological evaluations: According to the NBM standards, up to 25 different brain regions were analyzed and neuropathological reports were drawn in accordance with the National Institute on Aging-Alzheimer’s Association guidelines for the neuropathologic assessment of Alzheimer’s disease [50], the guidelines for the staging of Alzheimer disease-associated neurofibrillary pathology using paraffin sections and immunocytochemistry by Braak et al. [13], the CERAD (Consortium to Establish a Registry for Alzheimer’s Disease) guidelines [95] and the phases of amyloid beta deposits in the human brain by Tahl et al. [126]. Donors with any indications for bleedings, infarctions, stroke, tumors or sepsis were not considered for this study.

### Separation of neuronal and glia nuclei

Human postmortem occipital cortex (Brodmann area 17–19) neuronal and glia nuclei were separated by fluorescence-assisted isolation of immunolabeled nuclei using a NeuN- (RBFOX3-) specific antibody as described elsewhere [75, 93]. Thereby, most neuronal cell-types throughout the nervous system show positivity for NeuN, only distinct neuronal cell-types such as cerebellar Purkinje cells, olfactory bulb mitral cells and retinal photoreceptor cells stain negative [128]. Briefly, 600 mg of cortex tissue was accurately dissected, cut into small pieces after removing of leptomeninx and dissolved in 11 ml tissue lysis buffer (TLB) (10 mM Tris-HCl (pH 8), 0.1 mM EDTA, 3 mM Mg(Ac)$_2$, 5 mM CaCl$_2$, 1 tablet proteinase inhibitor cocktail, 0.1 mM PMFS, 0.16 mM DTT, 0.1% Igepal, 0.32 M sucrose). After homogenization by douncing, samples were transferred into ultracentrifugation tubes. Carefully 20 ml of nuclei separation buffer (NSB) (10 mM Tris-HCl (pH 8), 3 mM Mg(Ac)$_2$, 1 tablet proteinase inhibitor cocktail, 0.1 mM PMFS, 0.16 mM DTT, 1.8 M sucrose) were pipetted onto the bottom of the tubes. Separation of nuclei was performed using a Hitachi Sorvall Discovery 90SE ultracentrifuge with a Sorvall TH-641 rotator (Sorvall, Breda, Netherlands) at 24,400 rpm (1 h, $4\,^{\circ }\hbox {C}$). Nuclei pellets were resuspended in 4 ml ice-cold nuclei protection buffer (NPB) (2.7 mM KCl, 137 mM NaCl, 1.8 mM KH$_2$PO4, 10 mM $\hbox {Na}_2\hbox {HPO}_4$, 1 tablet proteinase inhibitor cocktail) and incubated on ice for 20 min. Neuronal nuclei were labeled by incubation for 1 h at RT with 6 $\upmu$l mouse-anti-NeuN-antibody (1 mg/ml) (Millipore, Eschborn, Germany) followed by addition of 6 $\upmu$l fluorescence-labeled secondary antibody (Alexa Fluor 546 goat-anti-mouse (2 mg/ml), Invitrogen, Darmstadt, Germany), 3 $\upmu$l DAPI (4’,6-Diamidin-2-phenylindol, Roth, Karlsruhe, Germany) and another incubation for 1 h in the dark. After centrifugation (8000 rpm, 30 s) pellets were resuspended in 3 ml ice-cold PBS and used for flow-cytometrical separation of neuronal (NeuN+ / DAPI+) and glial nuclei (NeuN−/DAPI+) on a FACSAria II device (BD Biosciences, Frankin Lakes, USA). Purity of nuclear fractions was $>98\%$ as checked by re-FACS and high-throughput fluorescence microscopy (IN Cell Analyzer 2000, GE Healthcare, Chalfont St. Giles, UK).

### Infinium 450k bead chip methylation profiling and data analysis

DNA extraction was performed using the QIAamp DNA Micro Kit (Qiagen, Hilden, Germany) according to the manufacturer’s protocol. DNA was dissolved in 90 $\upmu$l nuclease-free water and centrifuged at 14,000 rpm for 1 min. DNA was stored at $-80\,^{\circ }\hbox {C}$ for further use. Bisulfite treatment was performed using 1 $\upmu$g of genomic DNA in the EZ DNA Gold Methylation Kit (Zymo, Irvine, USA) according to manufacturer’s protocol, except that the final elution volume was 12 $\upmu$l. To assess DNA methylation signatures of 485,512 CpGs, we used Infinium HumanMethylation450 BeadChips (Illumina, San Diego, USA) according to the manufacturer‘s protocol. Arrays were scanned on an Illumina HiScan device.

### Data analysis

For methylation data processing and analysis we essentially followed recent guidelines on 450k data analysis [72]. Briefly, we performed quantile normalization and background subtraction using the R package minfi [1] and batch effect correction with Combat [57] from the sva package [71]. Methylation calls were generated as beta values, ranging from 0 to 1, representing 0% and 100% methylation, respectively. We removed probes with a detection *p* value $> 0.01$ in any samples. All samples had a probe call rate $>99\%$ and showed very high correlations ($R^2 = 0.99$) within cell-types (Additional file 1: Fig. S13). Data from the study of Guintivano et al. [35] were downloaded from NCBI GEO database (GSE41826; only the 23 healthy Caucasian controls were used) and processed the same way as our own data. Two additional external brain tissue datasets (GSE59685 and GSE80970) were downloaded and used for detailed comparisons. Data was downloaded as processed beta values (dasen normalized, no background subtraction) and corrected for batch effects with Combat. For the cell-type-specific analyses on aging or Braak stage progression, we merged our sorted dataset with data from Guintivano and colleagues to obtain a combined dataset of 478,416 CpGs and 108 sorted samples (78 controls and 30 AD samples). Then we run a principal component analysis (Additional file 1: Fig. S2) and correlated the principal components (PC) to known variables. PC1 showed best correlation with array batches ($p = 2.86 * 10^{-13}$), PC2 with cell-type ($p = 8.8 * 10^{-97}$), PC3 with sex ($p = 4.29 *10^{-43}$) and PC4 with age ($p = 1.08 * 10^{-17}$). We applied multiple linear regression models to find associations of DNA methylation and Braak stages with correction for confounding factors age and sex. We also corrected for technical batch effects in our model by including principal components 1 (PC1) from a principal component analysis as it shows high correlation to array batches :

1 $$\begin{aligned} CpG = \beta {_0} + \beta {_1} Braak + \beta {_2} Age + \beta {_3} Sex + \beta {_4} PC1 \end{aligned}$$

Since epigenomes are highly cell-type-specific we run this model separately for neuronal and glia samples. Then we performed a meta-analysis to combine results from the primary analyses using Fisher’s method with:

2 $$\begin{aligned} p = {\chi }^2 \left( -2 * \sum (\hbox {log} (p\;\hbox {values}))\right) \end{aligned}$$

Power analyses are difficult for EWAS as numerous markers have shown high correlations [72] and established methods to correct for multiple testings are likely to be too stringent. Therefore, if not noted otherwise we worked with nominal *p* values as done in other recent EWAS [27, 82]. Nevertheless, we additionally provide FDR adjusted *p* values in all relevant result tables. GO enrichment analyses were performed with the GOrilla tool using gene symbols according to Illumina annotation. KEGG pathway analyses were performed by converting relevant CpG identifiers to associated entrez-IDs according to Illumina annotation. Then, we used the R Package Pathview [85] for mapping to KEGG pathways. For comparative analyses we grouped samples into three groups with low (Braak stages 0, I, II; average age: 48.3), medium (III, IV; 85) or high (V, VI; 77.9) Braak staging.

### Estimation of neuronal cell proportions in brain tissue data

For the estimation of neuronal proportions in tissue data, we used the Houseman et al. surrogate measures of cell mixtures approach. As reference markers, we used the top-ranking 600 CpGs from the comparison (Student’s *t* test) of neuron and glia healthy control samples. Cell proportions were then calculated for each tissue sample with the ‘projectCellType’ function from the minfi R-software package.

### Immunohistochemical staining

Immunohistochemical staining to assess AD classification was performed by investigation of up to 25 different brain regions according to the NBM protocols and formalin-fixed and paraffin-embedded. Thereby the extent of neurofibrillary tangle pathology was assessed by immunohistochemical staining of hyperphosphorylated tau (AT8) according to the guidelines published by Braak et al. and classified to stage 0 to VI [13]. Additionally, we performed stainings for DAPI, SORL1 and FOXP1 of occipital cortex regions (BA 17-19). Pictures were made using an Olympus BX50 microscope and a $20\times$ objective (Olympus, Tokyo, Japan) as well as a Color View III camera (Soft Imaging System, Münster, Germany). We quantified signals from several representative cases where FFPE-tissue of target region was available (three pictures for each sample, Additional file 2: Table S3). Statistical evaluation was done using ANOVA and Newman-Keuls post hoc test.

### Validation of methylation profiles using NGS

Validations of 450k results were done by deep sequencing of bisulfite amplicons using a subset of 28 samples. For PCR amplicon design 16 regions were selected (Additional file 2: Table S20). The amplicons were designed to overlap or be located close to CpGs featured on 450k platform. PCRs were set up in 30 $\upmu$l reactions using 3 $\upmu$l of $10\times$ Hot Fire Pol Buffer (Solis BioDyne, Tartu, Estonia), 4 $\upmu$l of 10 mM d’NTPs (Fisher Scientific, Pittsburgh, USA), 2.25 $\upmu$l of 25 mM MgCl$_2$ (Solis BioDyne, Tartu, Estonia), 0.6 $\upmu$l of amplicon specific forward and reverse primer (10 $\upmu$M each), 0.3 $\upmu$l of Hot FirePol DNA Polymerase (5 U/$\upmu$l ; Solis Biodyne, Tartu, Estonia), 1 $\upmu$l of bisulfite DNA and 18.25 $\upmu$l of double distilled water. PCRs were run in an ABI Veriti thermocycler (Life Technologies, Karlsbad, USA) using the following program: 95 $^{\circ }\hbox {C}$ for 10 min, then 40 cycles of 95 $^{\circ }\hbox {C}$ for 1 min, 2.5 min of 56 $^{\circ }\hbox {C}$ and 40 sec at 72 $^{\circ }\hbox {C}$, followed by 7 min of 72 $^{\circ }\hbox {C}$ and hold at 4 $^{\circ }\hbox {C}$. PCR products were cleaned up using Agencourt AMPure XP Beads (Beckman Coulter, Brea, USA). All amplified products were diluted to 4 nM. Next, sequencing adapters compatible to the MiSeq platform (Illumina, San Diego, USA) were attached by PCR: a typical 50 $\upmu$l reaction contained 25 $\upmu$l of the DNA pool, 5 $\upmu$l of $10\times$ HotStartTaq buffer (Qiagen, Hilden, Germany), 4 $\upmu$l of 10 mM d’NTPs, 2 $\upmu$l of 25 mM MgCl$_2$, 2.5 $\upmu$l of 10 $\upmu$M universal-primer, 2.5 $\upmu$l of 10 $\upmu$M index-primer (unique for each sample), 0.6 $\upmu$l of HotStartTaq polymerase (Qiagen, Hilden, Germany) and 8.6 $\upmu$l of double distilled water. The reactions were incubated for 15 min at 97 $^{\circ }\hbox {C}$, followed by 5 cycles of 97 $^{\circ }\hbox {C}$ (30 s), 60 $^{\circ }\hbox {C}$ (30 s) and 72 $^{\circ }\hbox {C}$ (30 s). After another AMPURE bead-based cleanup step, samples were quantified by a Qubit High Sensitivity Assay (Life Technologies, Karlsbad, USA) and diluted to 10 nM. Finally, all samples were pooled and loaded on an Illumina MiSeq sequencing machine. Amplicons were sequenced $2 \times 250$ bp (paired end) involving a MiSeq reagent kit V2 chemistry (Illumina, San Diego, USA). The raw sequencing data was quality checked using FastQC (v0.10.3) and trimmed for adapters and low quality bases using the tools cutadapt (v1.3) and Trim Galore! (v0.3.3). Paired reads were joined using the FLASh tool. Next, reads were sorted in a two-step procedure by (1) the NGS barcode adapters to assign Sample ID and (2) the initial 15 bp to assign amplicon ID. Subsequently, the sorted data was loaded into the BiQAnalyzer HT software 22 using the following settings: the analyzed methylation context was set to ‘CG’, minimal sequence identity was set to 0.8 and minimal conversion rate was set to 0.95. The filtered high quality reads were then used for methylation calls of the respective CpGs. For non-CpG methylation analysis the methylation context was set to “C” and minimal sequence identity was set to 0.7.

### Public datasets

Public DNA methylation data from sorted frontal cortex brain samples were downloaded from GEO accession GSE41826 [35]. Only data from 29 Caucasian control donors were used and merged with our own sorted data. Two public brain tissue datasets were downloaded from GEO (GSE59685 [82] and GSE80970 [121]) and—in addition to our own tissue cohort—used for the comparison of sorted and bulk brain tissue data. Data from sorted prefrontal cortex brain samples (NeuN-positive only) under GEO accession GSE98203 [61] were downloaded (fully processed methylation values for 28 healthy control samples) and used for replication of top aging markers in neurons.

## Ethics approval and consent to participate

All procedures performed in studies involving human participants were in accordance with the ethical standards of the institutional and/or national research committee and with the 1964 Helsinki Declaration and its later amendments or comparable ethical standards.

## Additional files

**Additional file 1.** Supplementary Figures.

**Additional file 2.** Supplementary Tables.

## Abbreviations

5-caC: 5-carboxymethylcytosine
5-fC: 5-formylcytosine
5-hmC: 5-hydroxy-methylcytosine
5-mC: 5-methylcytosine
ACh: acetylcholine
AD: Alzheimer’s disease
ADAM17: ADAM metallopeptidase domain 17
ANK1: ankyrin 1
APP: amyloid beta precursor protein
ARSG: arylsulfatase G
BA: Brodmann area
BIN1: bridging integrator 1
CDH23: cadherin-related 23
CERAD: consortium to establish a registry for Alzheimer’s disease
CLU: clusterin
CNS: central nervous system
ct: cell-type
CTCF: CCCTC-binding factor
CTRL: control
CUX1: cut like homeobox 1
CXXC5: CXXC finger protein 5
DIP2C: disco-interacting protein 2 homolog C
DMCG: differentially methylated CpG
DNA: deoxy-ribonucleic acid
EC: entorhinal cortex
ELOVL2: fatty acid elongase 2
EWAS: Epigenome-wide association study
FACS: fluorescence-activated cell sorting
FAM53B: sequence similarity 53 member B
FAM83H: family with sequence similarity 83 member H
FC: frontal cortex
FFPE: formalin-fixed and paraffin-embedded
FHL2: four and a half LIM domains 2
FOXP1: forkhead box P1
GABA: gamma-aminobutyric acid
GEO: gene expression omnibus
GNG7: G protein subunit gamma 7
GO: gene ontology
HDAC4: histone deacetylase 4
HLA-DPB1: major histocompatibility complex, class II, DP beta 1
HOPX: HOP homeobox
HOXA3: homeobox A3
IL1RAPL1: interleukin 1 receptor accessory protein-like 1
INPP5A: inositol polyphosphate-5-phosphatase A
ITPK1: inositol-tetrakisphosphate 1-kinase
KEGG: Kyoto encyclopedia of genes and genomes
LRRC8B: leucine-rich repeat containing 8 VRAC subunit B
MAP2: microtubule-associated protein 2
MAPK: mitogen-activated protein kinase 1
MBP: myelin basic protein
MCF2L: MCF.2 cell line-derived transforming sequence like
NBM: neurobiobank Munich
NCOR2: nuclear receptor corepressor
NeuN: NeuN antigen
NGS: next-generation sequencing
RAI1: retinoid acid induced 1
PAX6: paired box 6
PCA: principal component analysis
PCR: polymerase chain reaction
PSEN1: presenilin 1
PSEN2: presenilin 2
PTPRN2: protein tyrosine phosphatase receptor type N2
RBFOX3: RNA binding Fox-1 homolog 3
RhoA: Ras homolog family member A
ROCK: Rho-associated coiled-coil containing protein
RPL13: ribosomal protein L13
S100B: S100 calcium binding protein B
SEC14L1: SEC14-like lipid binding 1
SORL1: sortilin-related receptor 1
STK32C: serine/threonine kinase 32C
SYNJ2: synaptojanin 2
SYNPO: synaptopodin
TC: temporal cortex
Tet: ten-eleven translocation
