# Supplementary material for: DNA methylation analysis on purified neurons and glia dissects age and Alzheimer’s disease-specific changes in the human cortex
Source: Epigenetics Chromatin. 2018 Jul 25;11:41. doi: 10.1186/s13072-018-0211-3 (PMC6058387; doi:10.1186/s13072-018-0211-3)
Supplement: Supplementary file 1 — Additional file 1. Supplementary Figures. [file 13072_2018_211_MOESM1_ESM.pdf]

Supplementary Figure 1

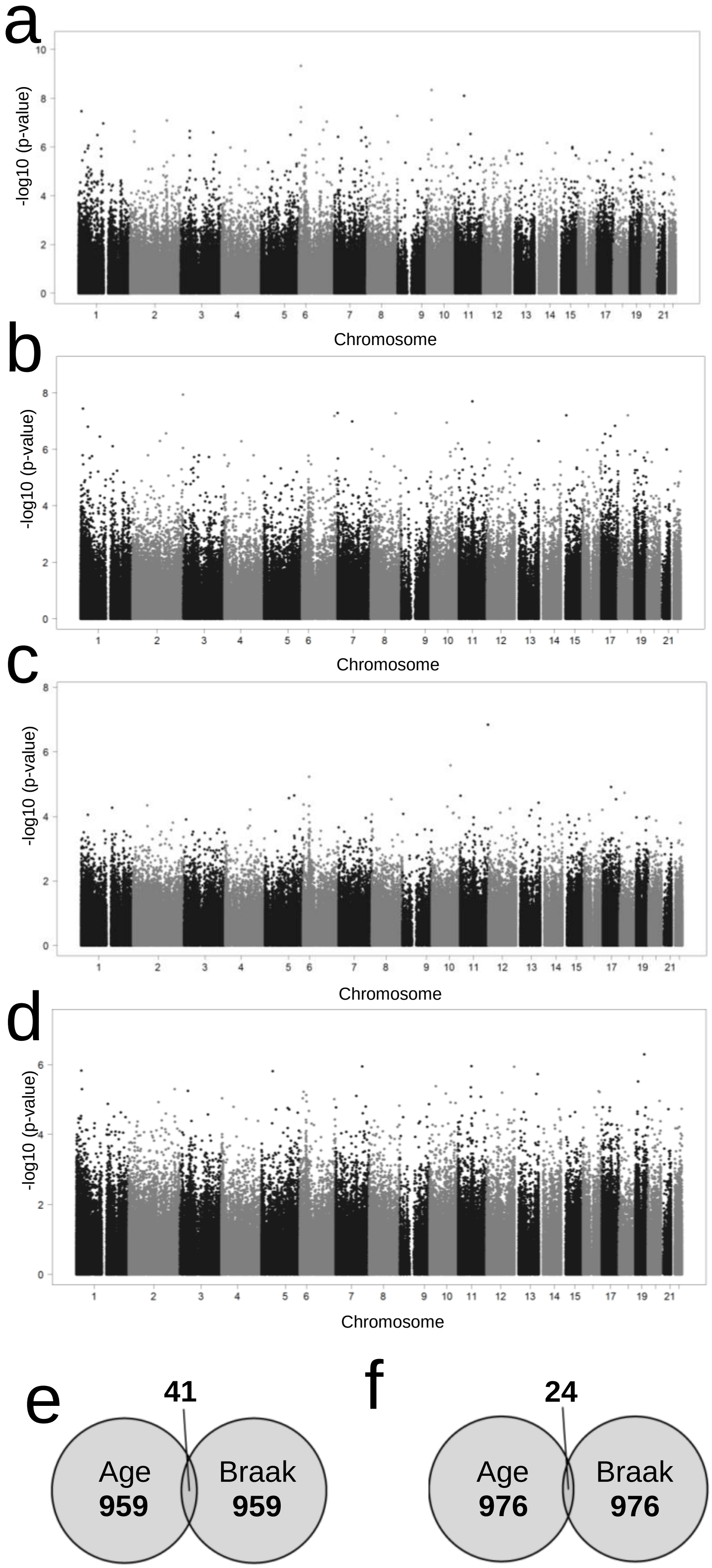

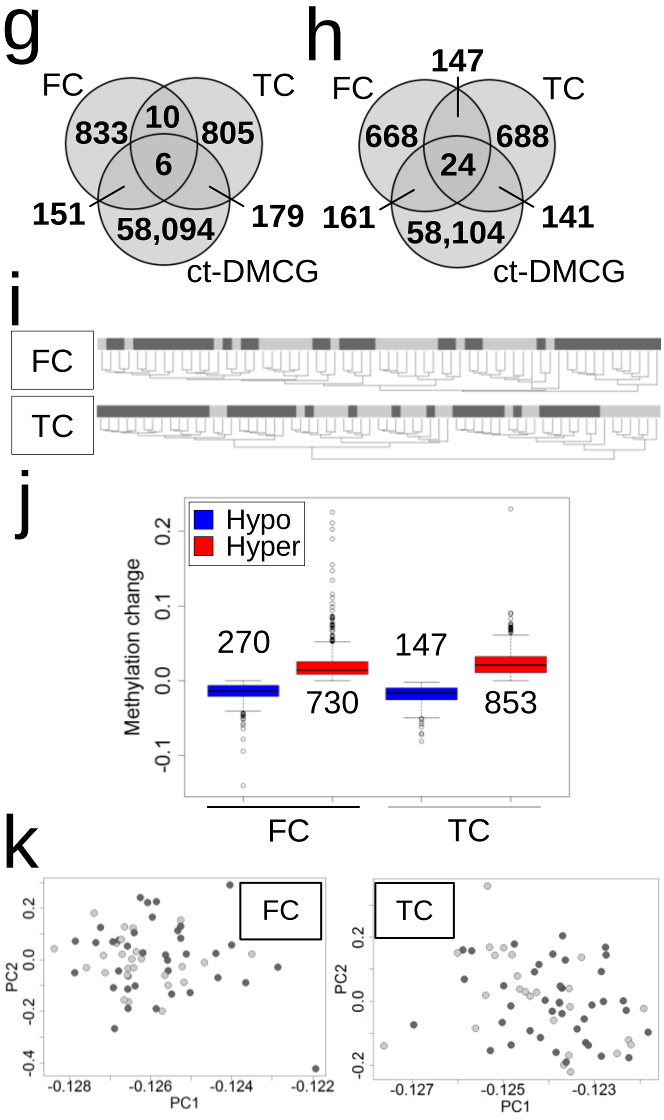

l

var1	var2	cor(FC)	p-value (FC)	cor(TC)	p-value(TC)
PC1	age	0.230	6.97E-02	0.129	3.05E-01
PC1	sex	-0.178	1.63E-01	-0.039	7.59E-01
PC1	diagnosis	0.310	1.35E-02	0.147	2.41E-01
PC1	array	-0.487	5.13E-05	0.356	3.56E-03
PC2	age	-0.079	5.36E-01	-0.053	6.74E-01
PC2	sex	0.142	2.68E-01	-0.071	5.75E-01
PC2	diagnosis	0.063	6.24E-01	-0.045	7.22E-01
PC2	array	-0.084	5.12E-01	-0.098	4.38E-01
PC3	age	-0.278	2.73E-02	0.580	4.19E-07
PC3	sex	0.013	9.18E-01	-0.053	6.78E-01
PC3	diagnosis	-0.042	7.42E-01	0.425	4.12E-04
PC3	array	-0.104	4.18E-01	-0.001	9.91E-01
PC4	age	0.614	8.65E-08	-0.064	6.10E-01
PC4	sex	-0.224	7.71E-02	0.170	1.75E-01
PC4	diagnosis	0.540	4.84E-06	-0.237	5.70E-02
PC4	array	-0.246	5.18E-02	0.014	9.14E-01

Supplementary Figure 2

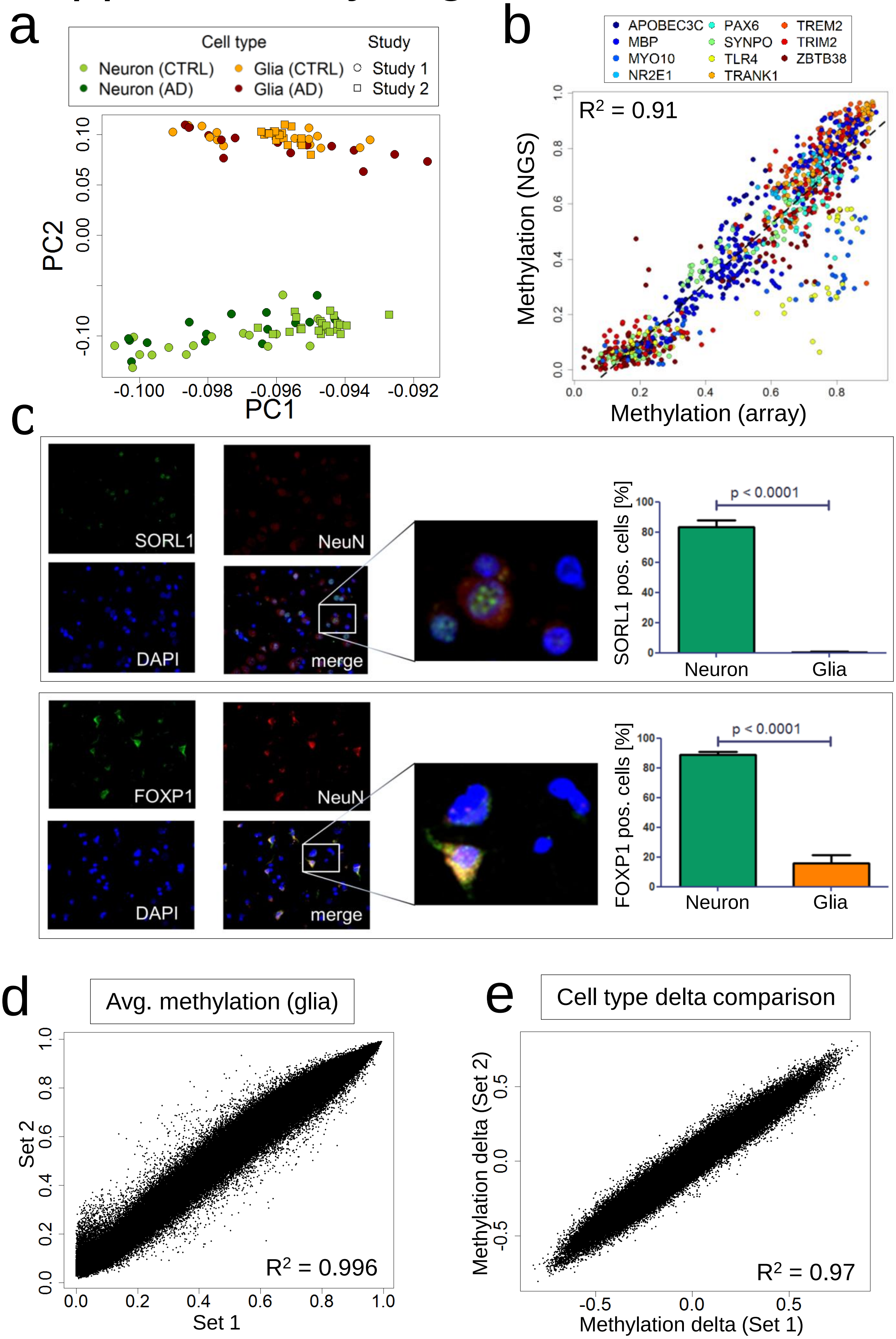

Supplementary Figure 3

a

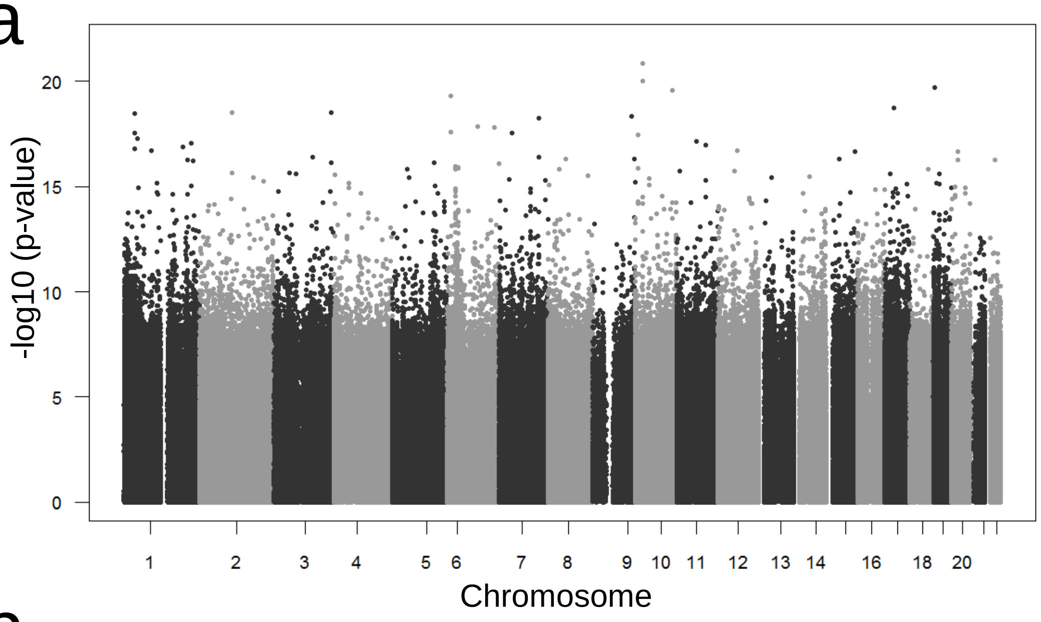

b

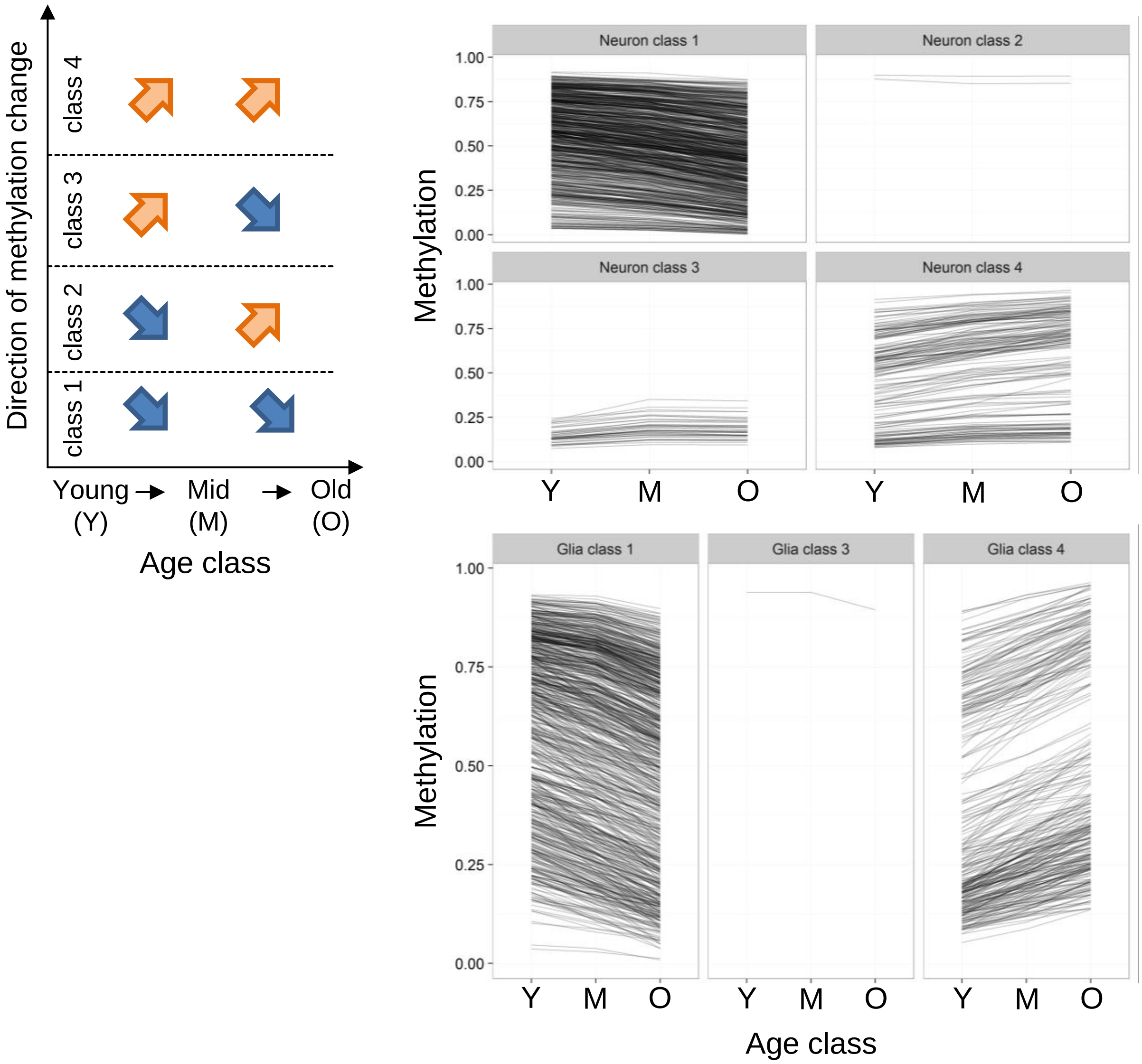

Supplementary Figure 4

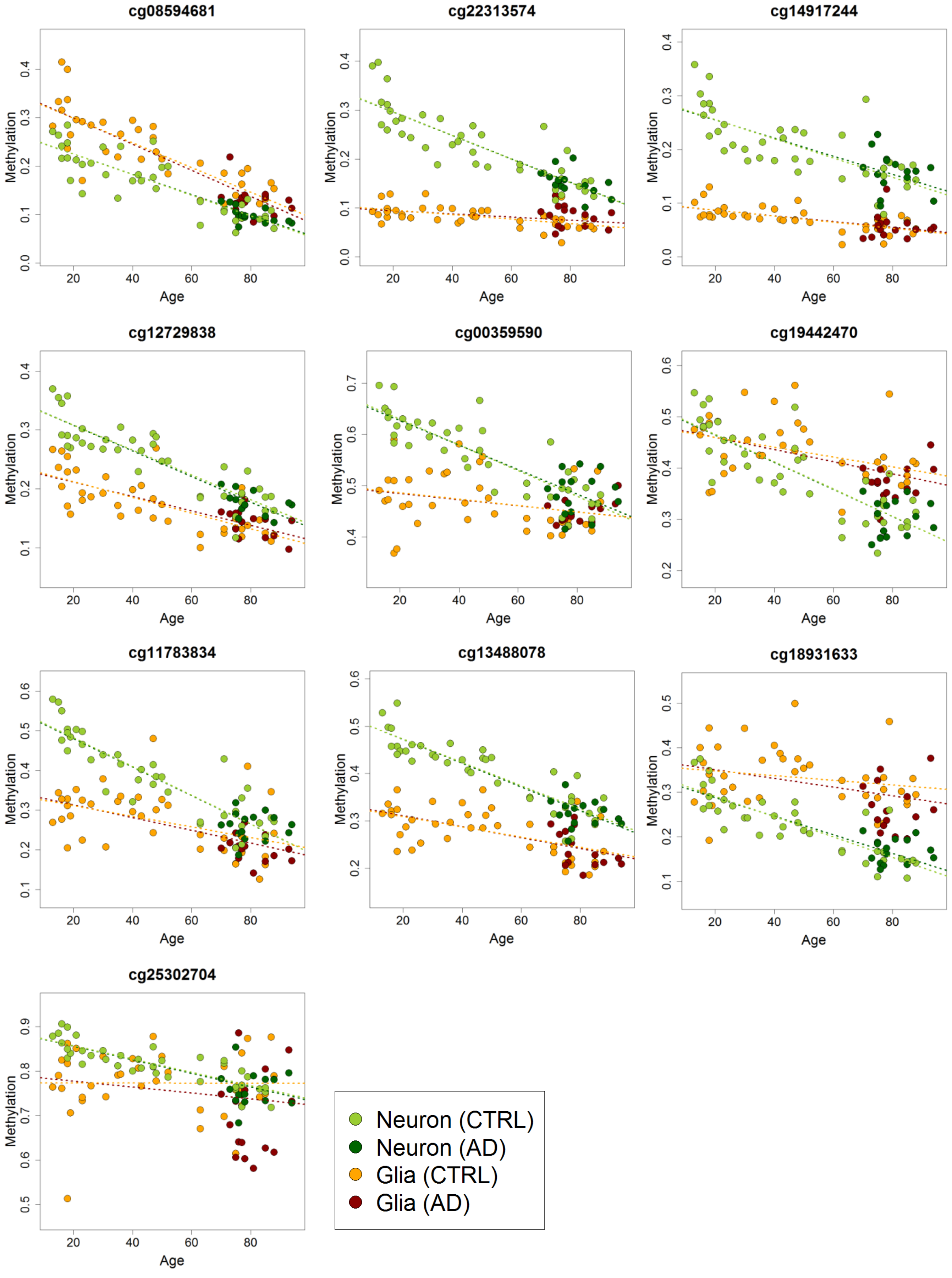

Supplementary Figure 5

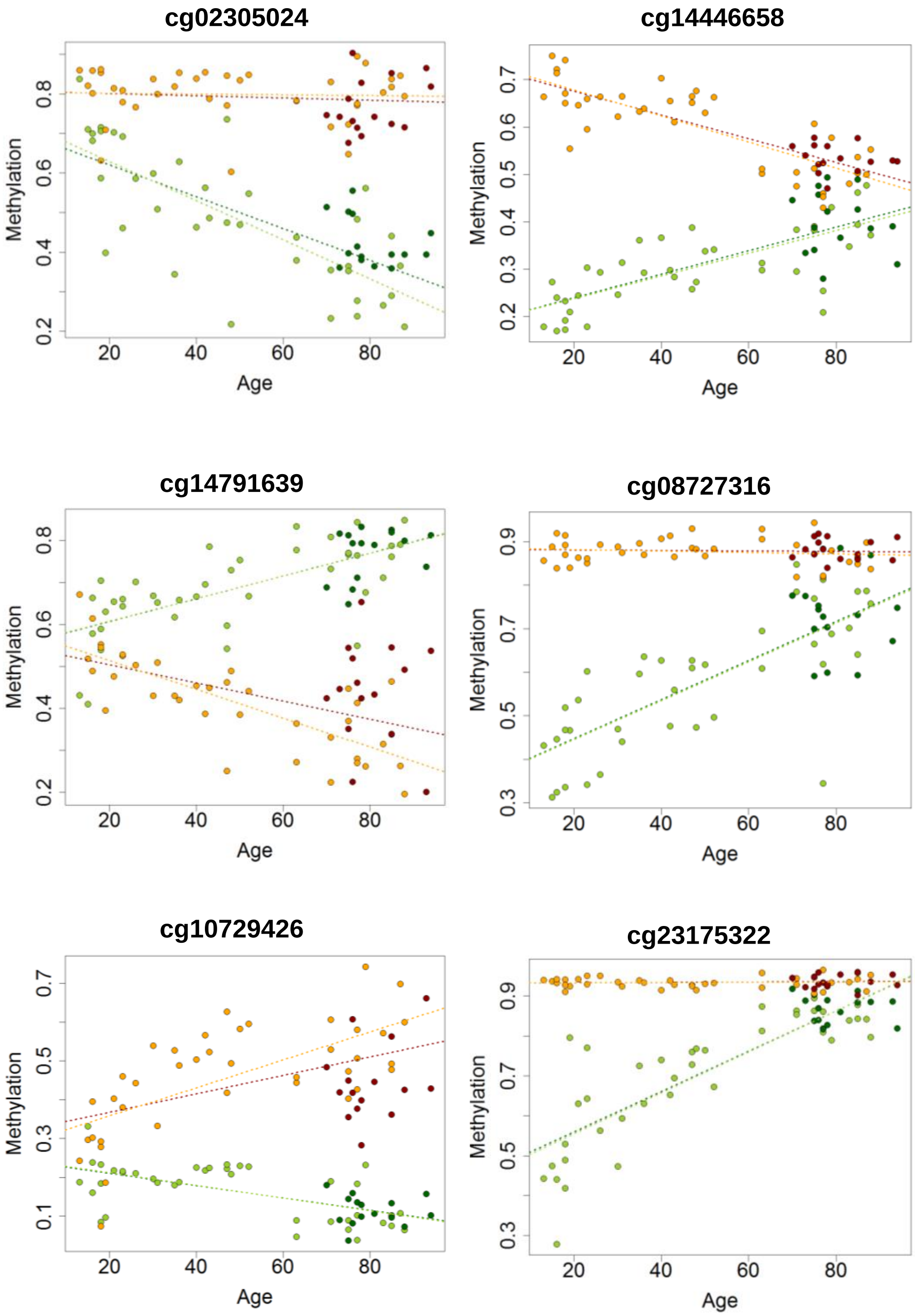

Supplementary Figure 6

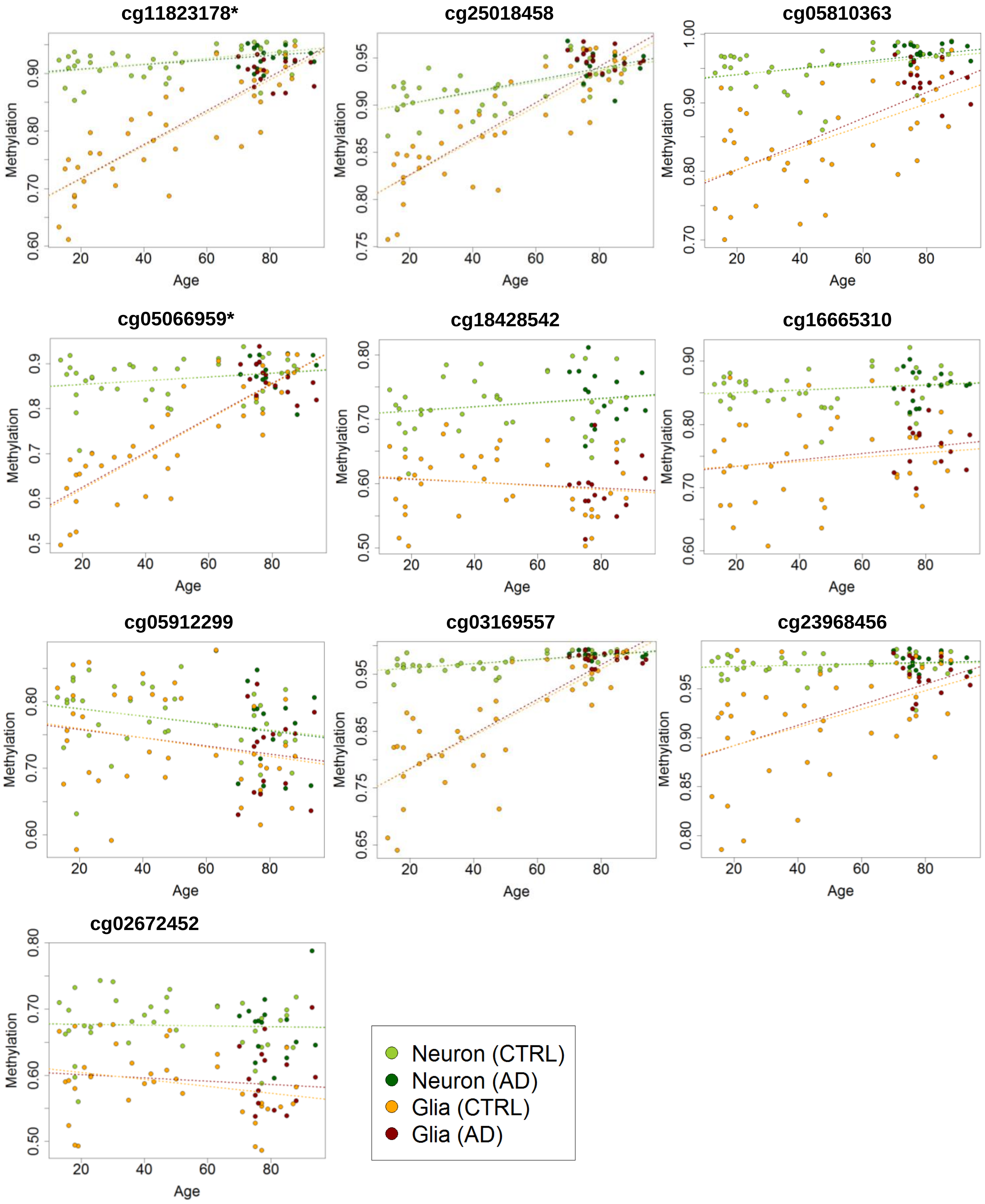

Supplementary Figure 7

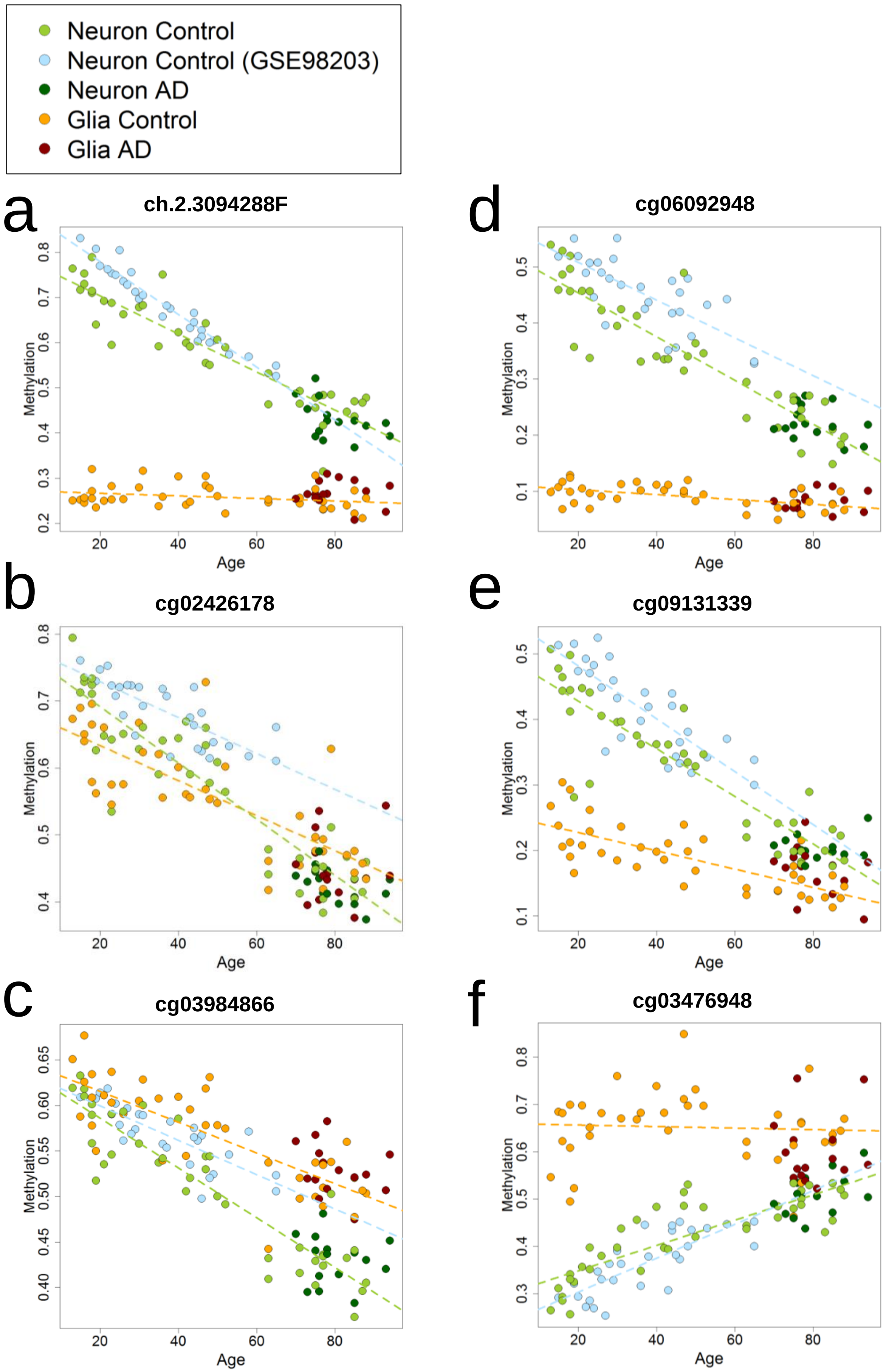

Supplementary Figure 8

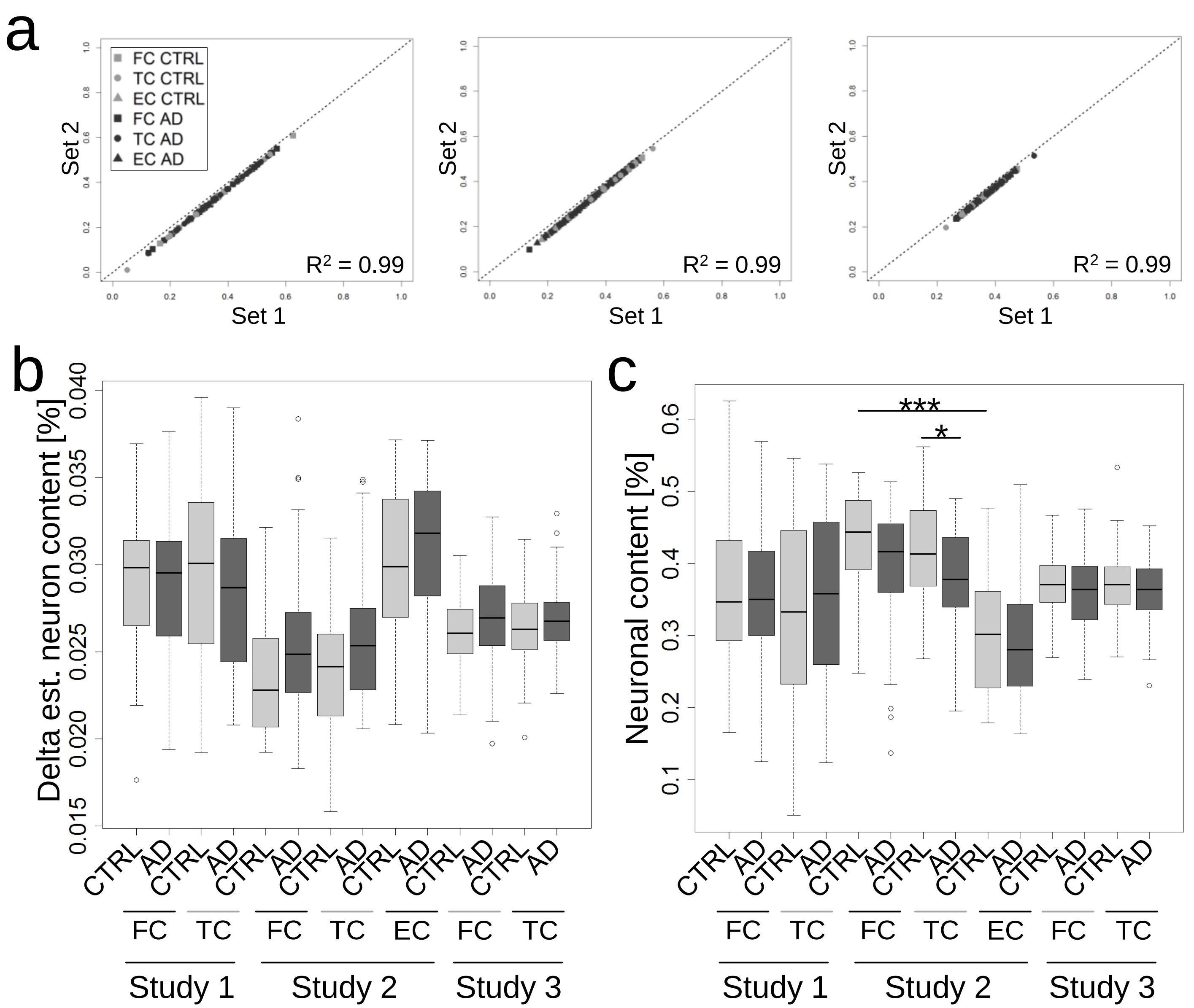

Supplementary Figure 9

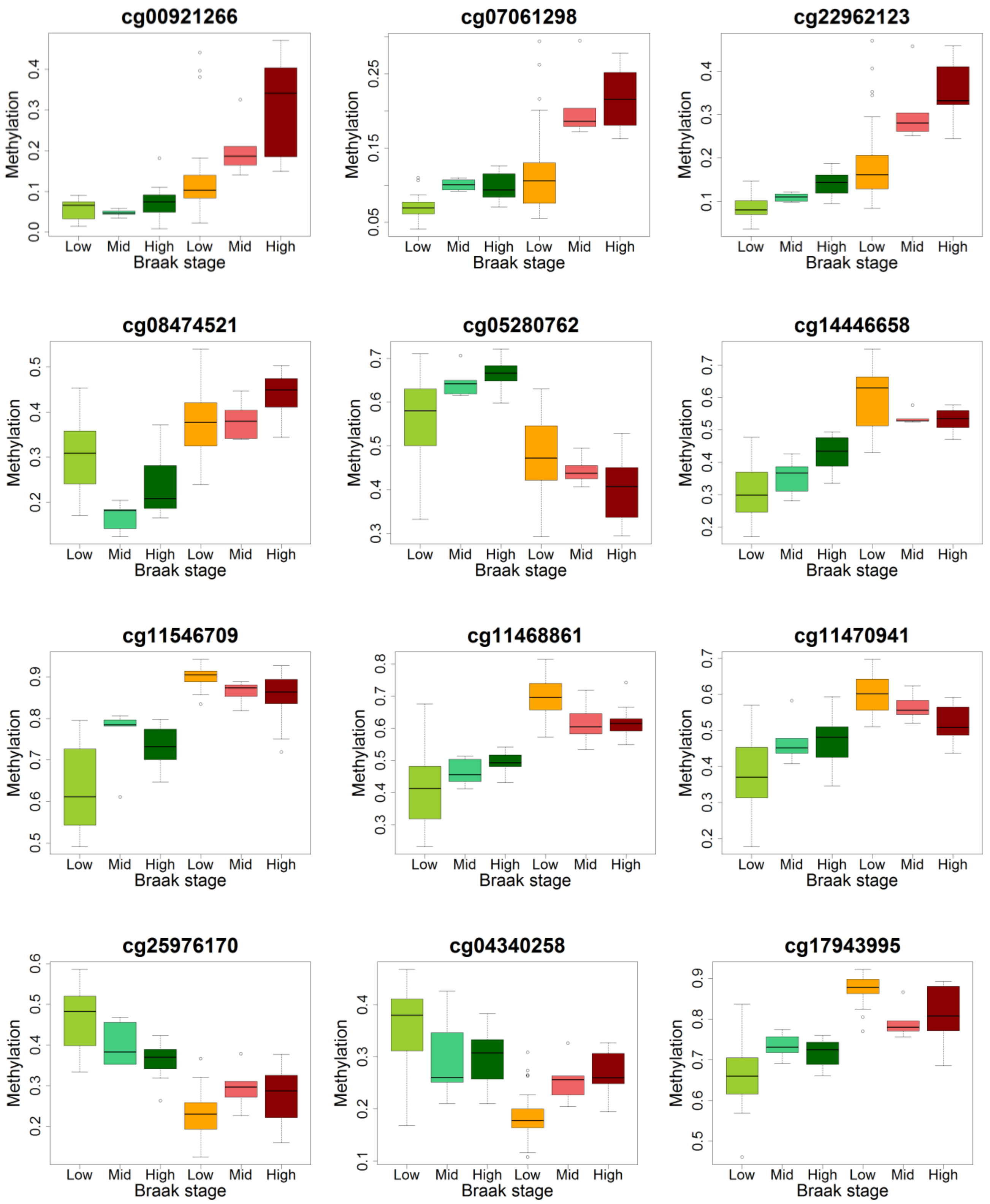

Supplementary Figure 10

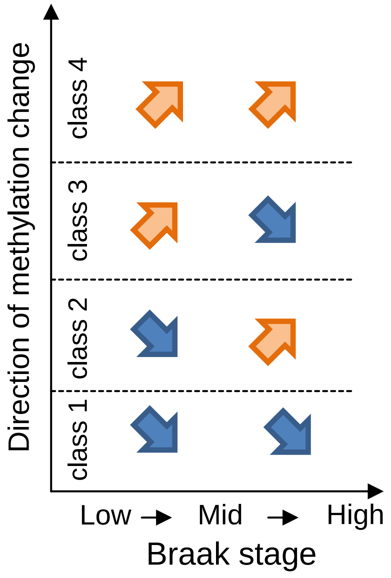

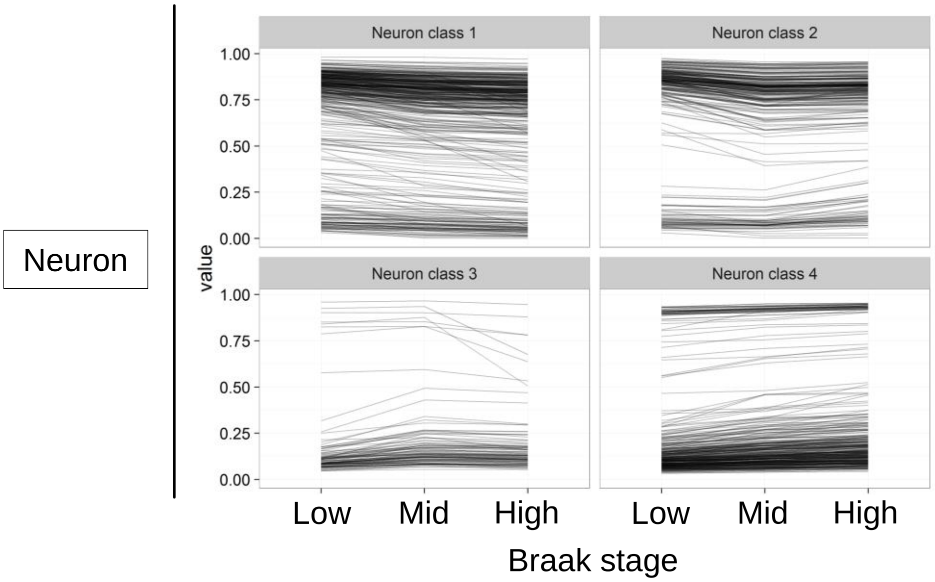

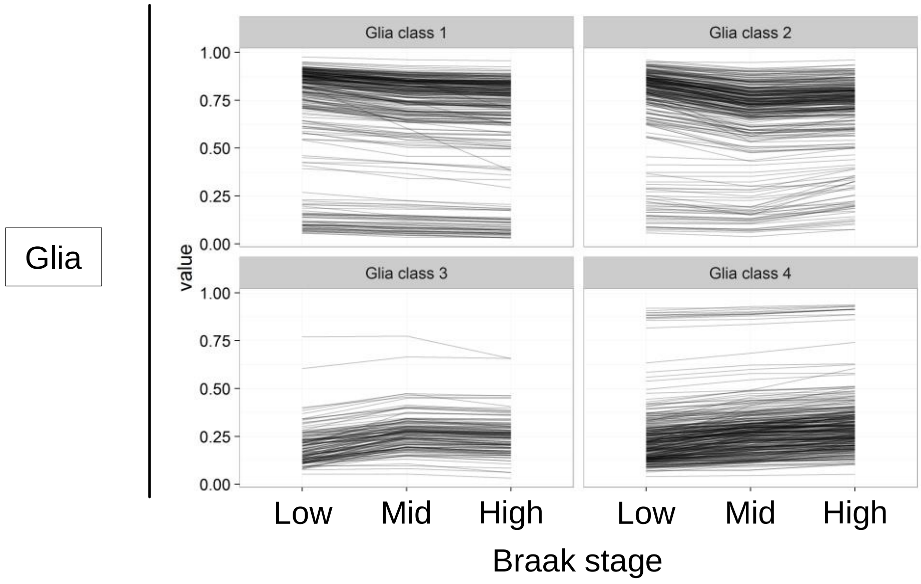

Supplementary Figure 11

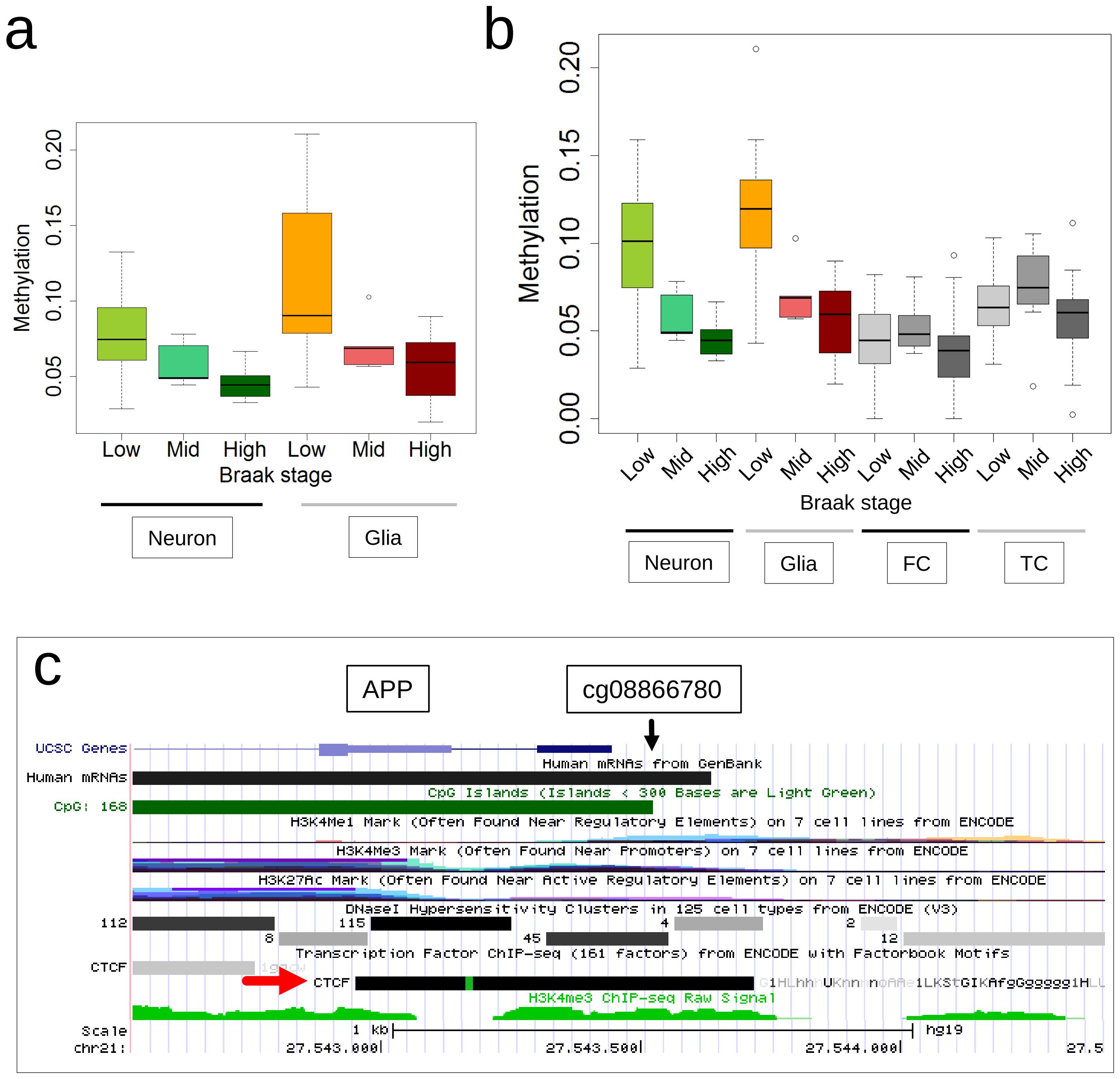

Supplementary Figure 12

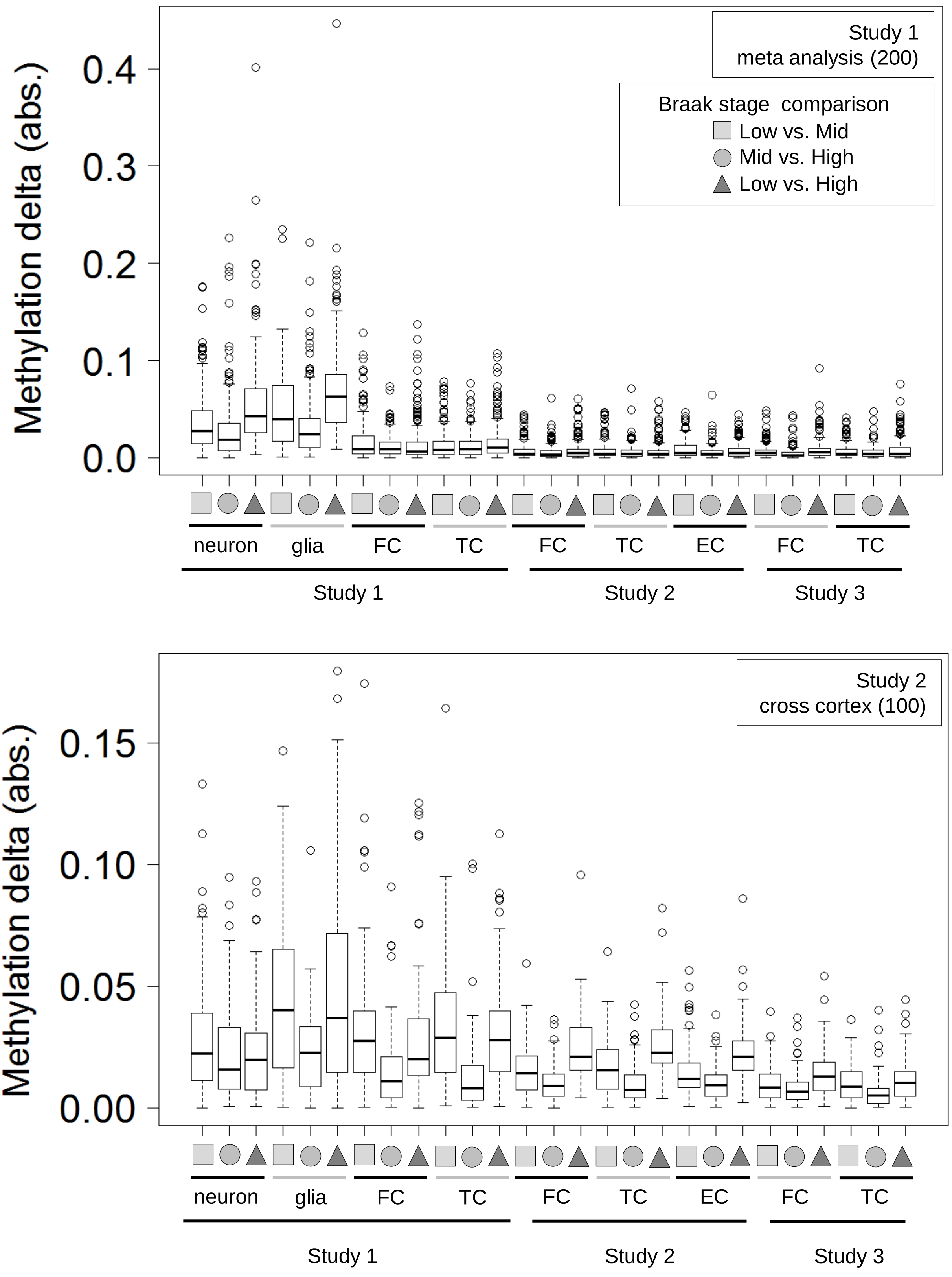

Supplementary Figure 14

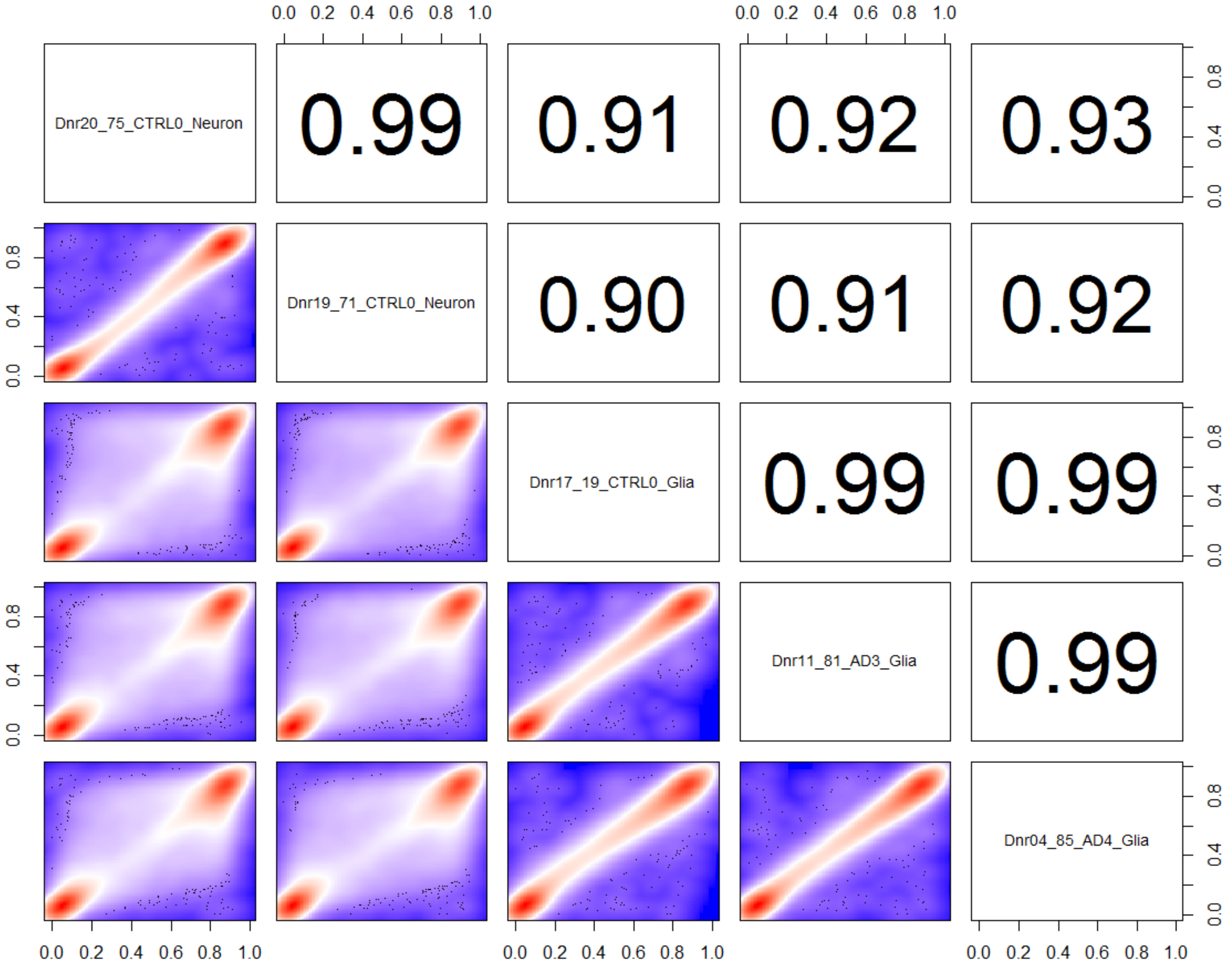

Supplementary Figure 1: Aging and Braak stage analyses in frontal and temporal cortex tissue samples. a): Genomic distribution of p-values for the aging analysis in FC. b): Genomic distribution of p-values for the aging analysis in TC. c): Genomic distribution of p-values for the Braak progression analysis in FC. d): Genomic distribution of p-values for the Braak stage progression analysis in TC. e): Overlap of top 1,000 Braak-DMCGs with aging-DMCGs in FC. f): Overlap of top 1,000 Braak-DMCGs with aging-DMCGs in TC. g): Overlap of top 1,000 ranking aging-DMCGs from FC or TC and ct-DMCGs. h): Overlap of top 1,000 Braak-DMCGs from FC or TC and ct-DMCGs. i): Cluster analysis using top 1,000 Braak-DMCGs from FC (left) or TC results in poor separation of AD and CTRL samples. j): Methylation changes between low and high Braak stages for the respective top 1,000 ranking Braak-DMCGs in frontal and temporal cortex, respectively. In both, frontal and temporal cortex most CpGs are hypermethylated in high Braak stage samples. k): Plots for principal components (PC) 1 and 2 from PCA in FC or TC; CTRLs are grey, AD samples in darkgrey. l): Correlation of individual PCs to known sample variables. Colors indicate low (red) or high (blue) values.

Supplementary Figure 2: Validation of cell type-specific methylation signatures and comparison to external data. a): PCA plot for autosomal CpGs using our own sorted data (study 1) and data from Guintivano et al. 2013 (GSE41826; study 2). b): High correlation for technical validation of 450k array data by deep next generation sequencing (NGS) of bisulfite amplicons. Eleven exemplary regions with profound methylation differences between neurons and glia were analyzed c): Exemplary immuno-histochemical validation of protein expression for the genes SORL1 (upper panel) and FOXP1 (bottom) that have significant ct-DMCGs. d) Similarity of glia profiles (CTRLs only) for our own study and data from study 2. e) Correlation plot illustrates high agreement for neuron vs. glia methylation delta on our study and study 2.

Supplementary Figure 3: Classification of top aging-DMCGs. a): Genomic p-value distribution for the aging analysis in glia. b): Dynamics of methylation changes for top 1,000 aging-DMCGs in CTRLs. The left sketch illustrates that samples were classified according to age into young (≤ 39 years, 'Y'), mid-age (40 - 69, 'M') and old (≥ 70, 'O') specimen and mean methylation was calculated. For each CpG the methylation changes from Y to M and M to O were used to assign the it to one of four possible classes (1 = down & down, 2 = down & up, 3 = up & down and 4 = up & up) and then plotted. For glia samples class 2 is not occupied and therefore omitted.

Supplementary Figure 4: Neuron-specific methylation dynamics upon aging at an alternative promoter of the *CLU* gene. Dark dashed lines represent regression lines using all neuron (green) or glia samples (red), bright lines are based on CTRLs while dark lines are based on all samples. Note for all CpGs there is a clear demethylation during aging for neuron samples and in many cases also for glia samples.

Supplementary Figure 5: Examples for divergent aging-dynamics in ct-DMCGs. Due to unbalanced or even reciprocal methylation dynamics in neurons and glia, these CpGs can defined as ct-DMCGs only at old (left) or young age (right). Dark dashed lines represent regression lines using all neuron (green) or glia samples (red), bright lines are only based on CTRLs. Legend see Supplementary Figure 6.

Supplementary Figure 6: Several top Braak stage CpGs identified in brain tissue EWAS exhibit pronounced and unbalanced aging dynamics in neuron and glia. Cell type-specific methylation levels plotted by age for the top 10 ranking Braak-DMCGs from a large tissue-based EWAS. Dark dashed lines represent regression lines using all neuron (green) or glia samples (red), bright lines are only based on CTRLs. For the ANK1 CpGs (*) and several other top ranking CpGs reported by GSE59685 pronounced cell type-specific methylation dynamics are observed over lifetime and are independent from disease status.

Supplementary Figure 7: Independent validation of aging-DMCGs. Top 5 (a-e) aging-DMCGs in neurons show similar methylation dynamics in an independent cohort of healthy NeuN sorted samples (GSE98203; n = 28). f): Example for a top ranking aging-DMCG (rank 26) with hypermethylation in late life. Glia samples are co-plotted to show cell type-specific aging dynamics. AD samples are co-plotted to illustrate absence of disease-related changes in these CpGs. Regression lines are based only on respective control samples (neurons, glia, neurons from GSE98203).

Supplementary Figure 8: Estimation of cell proportions in brain tissue data sets. a): Estimations for neuronal content in our own tissue data (left; study 1), data from Lunnon et al (mid; study 2) or data from GSE80970 (right; study 3). Estimation were once performed with the improved Houseman method using the top 600 ct-DMCGs and our own sorted reference data (set 1) or data from Guintivano et al. 2013 (set2). Estimations based on set 1 or set 2 correlate very well but are generally higher estimated by set 1. b): Deltas for neuron content estimation using set1 or set2. c): Comparison for estimated neuronal proportions across studies, tissues and diagnosis. For study 2, TC neuronal content in AD is significantly lower than in CTRLs (two-sided t test). (FC: frontal cortex; TC: temporal cortex; EC: entorhinal cortex; *: $p>0.05$; ***: $p<0.001$)

Supplementary Figure 9: Examples for Braak-DMCGs with unbalanced changes in neuron and glia. Methylation levels plotted by Braak stage and cell type. Greenish boxes are neuron and reddish boxes are glia samples.

Supplementary Figure 10: Methylation dynamics across Braak stages for top 1,000 Braak-DMCGs in neurons or glia. The sketch (top) illustrates how CpGs were classified in respect methylation changes upon Braak stage progression in neurons (middle panel) and glia (bottom).

Supplementary Figure 11: Braak stage-associated hypomethylation at the APP promoter. a): Hypomethylation at an APP promoter CpG (‘cg0886670’) during Braak stage progression in neurons and glia is age-independent. Only samples older than 64 years (n = 56) were used to generate this plot. b): Decrease of methylation levels at the APP promoter CpG ‘cg08866780’ over Braak stage progression in sorted but not in tissue samples. c): UCSC genome browser (http://genome.ucsc.edu/) snapshot illustrating the APP promoter region with ‘cg08866780’ (black arrow) which is located at CTCF binding site (red arrow). (FC: frontal cortex, TC: temporal cortex)

Supplementary Figure 12: Braak stage methylation differences are higher in sorted samples than in tissues. Absolute methylation differences between Braak stages for each sample cohort based on the top 200 CpGs from our meta analysis (top) or the top 100 cross cortex CpGs from the Lunnon study (bottom). (Study 1: our own data from sorted and bulk tissue samples; study 2: bulk tissue data from Lunnon and partners (GSE59685); study 3: bulk tissue data from GSE80970; FC = frontal cortex, TC = temporal cortex, EC = entorhinal cortex)

Supplementary Figure 13: High correlation between individual array samples. Pairwise plot of 5 randomly selected samples and 50,000 randomly selected probes from autosomal chromosomes. The upper panel gives the Pearson correlation value for the horizontally and vertically linked sample pair. Accordingly the lower panels displays smooth scatter plots of methylation values for the respective pair of samples. Colors from blue to white to red indicate increased density.
